# Oncogenic gene expression and epigenetic remodeling of cis-regulatory elements in ASXL1-mutant chronic myelomonocytic leukemia

**DOI:** 10.1038/s41467-022-29142-6

**Published:** 2022-03-17

**Authors:** Moritz Binder, Ryan M. Carr, Terra L. Lasho, Christy M. Finke, Abhishek A. Mangaonkar, Christopher L. Pin, Kurt R. Berger, Amelia Mazzone, Sandeep Potluri, Tamas Ordog, Keith D. Robertson, David L. Marks, Martin E. Fernandez-Zapico, Alexandre Gaspar-Maia, Mrinal M. Patnaik

**Affiliations:** 1 Division of Hematology, Mayo Clinic, Rochester, MN USA; 2 Epigenomics Program, Center for Individualized Medicine, Mayo Clinic, Rochester, MN USA; 3 Lawson Health Research Institute, University of Western Ontario, London, ON Canada; 4 Department of Laboratory Medicine and Pathology, Mayo Clinic, Rochester, MN USA; 5 Institute of Cancer and Genomic Sciences, University of Birmingham, Birmingham, UK; 6 Schulze Center for Novel Therapeutics, Division of Oncology Research, Mayo Clinic, Rochester, MN USA

**Keywords:** Myelodysplastic syndrome, Cancer genomics, Epigenomics, Gene regulation, Gene expression

## Abstract

Myeloid neoplasms are clonal hematopoietic stem cell disorders driven by the sequential acquisition of recurrent genetic lesions. Truncating mutations in the chromatin remodeler ASXL1 (ASXL1^MT^) are associated with a high-risk disease phenotype with increased proliferation, epigenetic therapeutic resistance, and poor survival outcomes. We performed a multi-omics interrogation to define gene expression and chromatin remodeling associated with ASXL1^MT^ in chronic myelomonocytic leukemia (CMML). ASXL1^MT^ are associated with a loss of repressive histone methylation and increase in permissive histone methylation and acetylation in promoter regions. ASXL1^MT^ are further associated with de novo accessibility of distal enhancers binding ETS transcription factors, targeting important leukemogenic driver genes. Chromatin remodeling of promoters and enhancers is strongly associated with gene expression and heterogenous among overexpressed genes. These results provide a comprehensive map of the transcriptome and chromatin landscape of ASXL1^MT^ CMML, forming an important framework for the development of novel therapeutic strategies targeting oncogenic cis interactions.

## Abstract (web summary)

‘Mutations in the chromatin remodeler ASXL1 (ASXL1^MT^) are associated with poor clinical outcome, however, their impact on chromatin dynamics remains unexplored. Here the authors use a multi-omics approach for chronic myelomonocytic leukemia (CMML) and investigate the transcriptome and chromatin landscape of ASXL1^MT^ CMML.

## Introduction

Chronic myeloid neoplasms are malignant clonal hematopoietic stem cell disorders driven by recurrent genetic events, with an inherent risk of transformation to acute myeloid leukemia (AML)^1,2^. Within myeloid neoplasms, chronic myelomonocytic leukemia (CMML) represents an attractive disease model since it is characterized by both myelodysplastic and myeloproliferative features, while retaining a relatively simple clonal composition^3^. CMML shares the typical repertoire of genetic driver lesions with other myeloid neoplasms and is particularly enriched in truncating mutations involving *ASXL1* (prevalence ~40%)^3^. The presence of truncating *ASXL1* mutations in CMML is associated with proliferative disease features, resistance to epigenetic therapies, and adverse outcomes^4–6^. Due to their independent prognostic significance, *ASXL1* mutations have been incorporated in all three contemporary molecularly integrated CMML-specific prognostic models^5,7,8^. Given the paucity of effective therapies for CMML, delineating the molecular mechanisms of *ASXL1*-mutant CMML (ASXL1^MT^) is of particular interest from a therapeutic standpoint.

The sum of evidence from mechanistic studies suggests that ASXL1 has a complex interactome, that truncating *ASXL1* mutations promote leukemogenesis by transcriptional up-regulation of leukemogenic drivers including posterior *HOXA* genes, and that these mutations recruit several effectors to alter the epigenome through histone modifications, increases in chromatin accessibility, and remodeling of enhancers^9–14^. However, to our knowledge, no studies have been able to extensively query the epigenome in primary patient samples. Given the complexity of human transcriptional regulation in vivo and the multitude of potential epigenetic mechanisms cooperating to regulate transcriptional activity, questions remain about the interplay of regulatory mechanisms in patients with CMML. To elucidate this interplay, we interrogated the genome, transcriptome, and epigenome of patients with *ASXL1*-wildtype (ASXL1^WT^) and ASXL1^MT^ CMML. In this work, we integrated somatic mutations, transcription, (hydroxy)methylation, histone modifications, and chromatin accessibility to reveal the complexity of the epigenetic landscape, the simultaneous presence of multiple regulatory mechanisms affecting drivers of leukemogenesis, and remodeling of the enhancer landscape as an important driver of intratumoral heterogeneity. These insights into the epigenetic landscape of ASXL1^MT^ CMML generated from primary patient samples are of considerable interest for the development of novel targeted therapeutic strategies for patients with ASXL1^MT^ CMML.

## Results

**Fig. 1:**
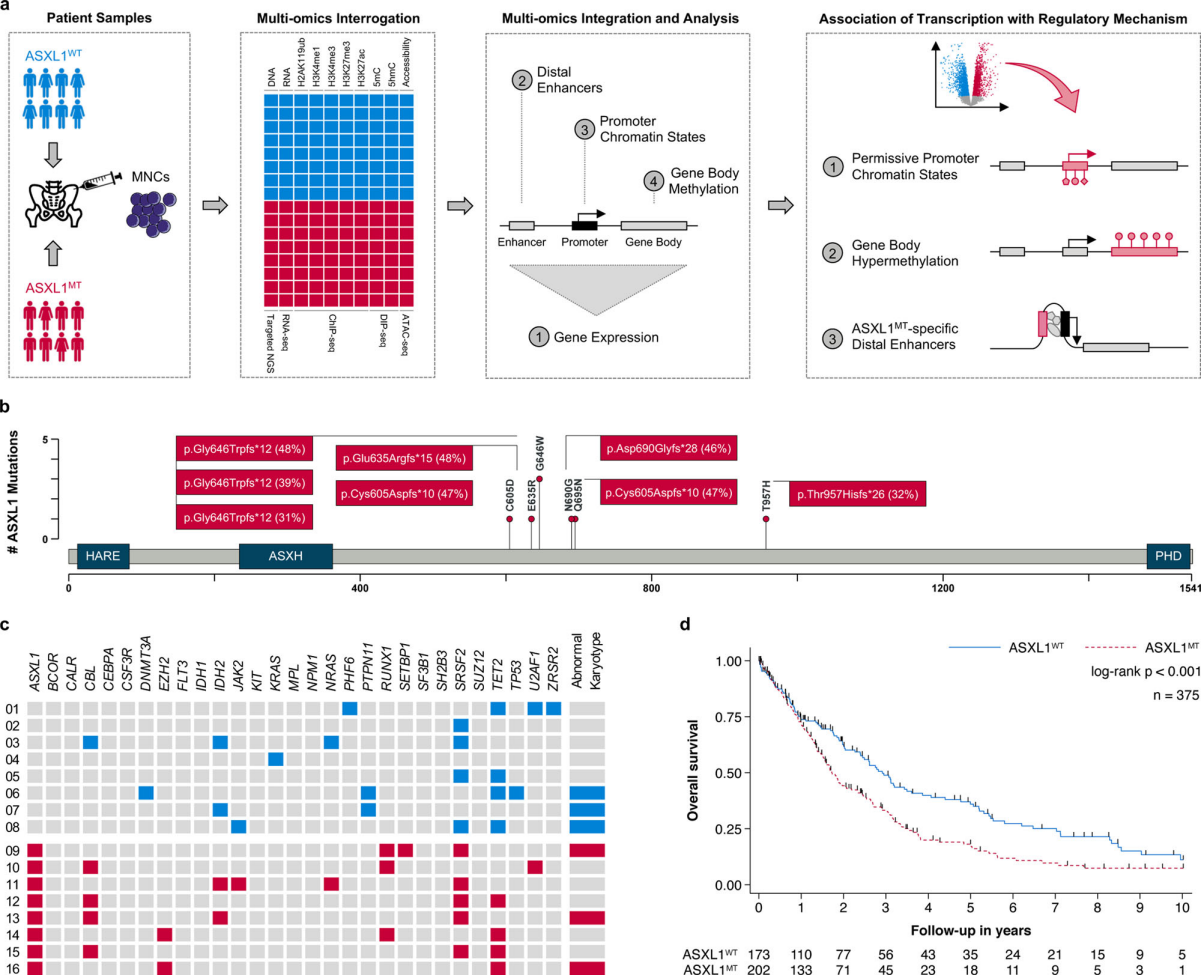
Truncating *ASXL1* mutations are of prognostic significance in chronic myelomonocytic leukemia and frequently co-occur with other mutations. **a** Flowchart showing the study design: An integrated multi-omics approach to discover ASXL1^MT^-specific epigenetic regulatory mechanisms associated with transcriptional up-regulation. **b** Lollipop plot showing that all mutations in *ASXL1* resulted in a frameshift preserving the HB1, ASXL, restriction endonuclease helix-turn-helix (HARE) and LXXLL motif alpha helical (ASXH) domain but not the plant homeodomain (PHD). All observed variant allele frequencies were compatible with heterozygosity. **c** Heatmap showing the spectrum of co-mutations, which included spliceosome components, chromatin regulators, modulators of DNA methylation, and cell signaling molecules. The prevalence of abnormal karyotypes and the burden of co-mutations were similar between ASXL1^MT^ and ASXL1^WT^ patients. **d** Kaplan–Meier plot showing overall survival estimates for the 375 patients with chronic myelomonocytic leukemia from which the 16 patients in this study were sampled from (median follow-up 18 months). The presence of truncating *ASXL1* mutations was associated with increased all-cause mortality in this patient population (median overall survival 1.72 years, 95% CI 1.51–2.19, *n* = 202 versus 2.92 years, 95% CI 2.39–3.61, *n* = 173; HR 1.54, 95% CI 1.19–1.98, *p* = 0.001). This association remained consistent after adjusting for age at diagnosis, sex, and the other factors of the Mayo Molecular Risk Stratification Model (HR 1.37, 95% CI 1.05–1.78, *p* = 0.019, *n* = 375)^8^. There were no violations of the proportional hazards assumption (*p* = 0.113). Source data are provided as a Source Data file.

To survey the epigenetic landscape of human CMML, we interrogated mutational spectrum, transcription, DNA methylation, histone modifications, and chromatin accessibility in ASXL1^MT^ (*n* = 8) and ASXL1^WT^ (*n* = 8) CMML (Fig. 1a). The clinical characteristics of the 16 patients with WHO-defined CMML included in this study are shown in Table 1 and Supplementary Data 2. All mutations in *ASXL1* resulted in a frameshift and were predicted to lead to a truncation of the protein’s plant homeodomain (Fig. 1b). The spectrum of co-mutations was consistent with previous observations and included spliceosome components, chromatin regulators, modulators of DNA methylation, and cell signaling molecules (Fig. 1c). Abnormal karyotypes were observed in the same number of patients and the burden of co-mutations was similar between the two groups (median number per group 3 versus 3, *p* = 0.508). This included several modulators of DNA methylation including *TET2*, *DNMT3A*, and *IDH2* (median number per group 1 versus 1, *p* = 0.699). As previously reported, G646W (c.1934dup) was the most prevalent *ASXL1* mutation and the observed variant allele frequencies of all *ASXL1* mutations were consistent with heterozygosity^15,16^. The presence of truncating *ASXL1* mutations was associated with increased all-cause mortality in the larger patient population seen at our institution (*n* = 375) from which the 16 patients is this study were sampled (Fig. 1d, Supplementary Fig. 1a, b). For additional information on patient, sample, and cell selection, please refer to *Methods* and Supplementary Fig. 1c.

**Table 1:** Clinical characteristics of 16 patients with CMML stratified by *ASXL1* genotype.

Parameter	Unit	ASXL1^WT^ (*n* = 8)	ASXL1^MT^ (*n* = 8)
Age at diagnosis	[years]	66 (12)	67 (13)
Male sex	[n (%)]	4 (50)	6 (75)
Hemoglobin	[g/dL]	12.3 (3.3)	11.6 (4.4)
Leukocytes	[x10^9^/L]	8.4 (9)	14.2 (23.9)
Neutrophils	[x10^9^/L]	3.2 (3.8)	6.5 (15.2)
Lymphocytes	[x10^9^/L]	2.3 (2.1)	1.9 (1.4)
Monocytes	[x10^9^/L]	2.0 (4.5)	2.8 (2.5)
Platelets	[x10^9^/L]	112 (60)	159 (151)
Peripheral blood blasts	[%]	0 (2)	0 (1)
Bone marrow blasts	[%]	4 (3)	2 (5)
Abnormal cytogenetics	[n (%)]	3 (38)	3 (38)
**Morphology (WHO 2016)**			
CMML-0	[n (%)]	5 (62)	4 (50)
CMML-1	[n (%)]	1 (13)	3 (37)
CMML-2	[n (%)]	2 (25)	1 (13)
**Mayo Prognostic Model**			
Low	[n (%)]	3 (37)	0 (0)
Intermediate	[n (%)]	2 (25)	4 (50)
High	[n (%)]	3 (38)	4 (50)
**Mayo Molecular Model**			
Low	[n (%)]	3 (37)	0 (0)
Intermediate-1	[n (%)]	2 (25)	0 (0)
Intermediate-2	[n (%)]	3 (38)	4 (50)
High	[n (%)]	0 (0)	4 (50)
Leukemic transformation	[n (%)]	4 (50)	5 (63)

Data are given as median (interquartile range) unless denoted otherwise.

### Truncating *ASXL1* mutations are associated with transcriptional up-regulation of genes involved in cell cycle progression and DNA replication

**Fig. 2:**
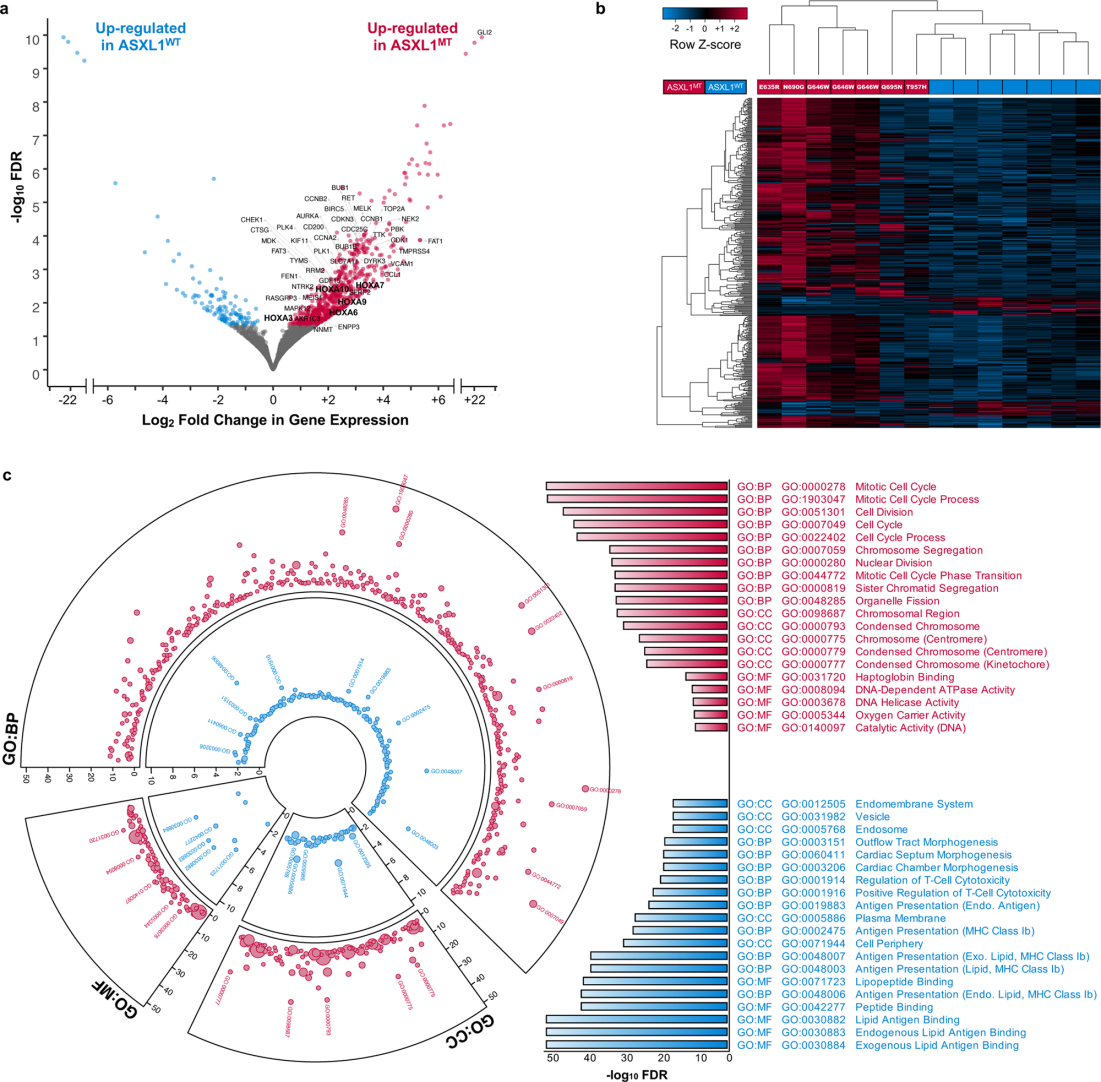
The transcriptome of ASXL1^MT^ CMML is characterized by transcriptional up-regulation of key mitotic pathways and leukemogenic driver genes. **a** Volcano plot showing a predominance of transcriptional up-regulation in ASXL1^MT^ CMML with a limited number of genes being down-regulated. Up-regulated therapeutic targets with therapeutic agents either being available or currently under development are labeled. Also labeled are the members of the posterior *HOXA* cluster including the leukemogenic driver *HOXA9* and its co-factor *MEIS1* (bold). **b** Heatmap showing the separation of ASXL1^MT^ and ASXL1^WT^ CMML by unsupervised hierarchical clustering of all differentially expressed genes with FDR < 0.010. **c** Circos plot showing the up-regulation of mitotic activity and down-regulation of MHC class I mediated antigen presentation and cytotoxic T-cell activity in ASXL1^MT^ CMML (red: up-regulated in ASXL^MT^ CMML, blue: up-regulated in ASXL^WT^ CMML). Bar graph showing the top hits in each gene ontology category (GO: Gene Ontology; BP: Biological Process; MF: Molecular Function; CC: Cellular Compartment). Axes represent the statistical significance of the gene ontology terms and the size of the markers is proportional to the number of genes per cluster.

To define the gene expression profile associated with truncating *ASXL1* mutations, we performed differential gene expression analysis (829 differentially expressed genes, FDR < 0.050, Fig. 2a, Supplementary Data 3, Supplementary Fig. 2a–c). There was a predominant up-regulation of transcriptional activity (707 genes) among ASXL1^MT^ patients. Of the 707 up-regulated genes in ASXL1^MT^ CMML, 217 were considered therapeutic targets (Fig. 2a, Supplementary Data 4)^17^. Unsupervised hierarchical clustering separated ASXL1^MT^ from ASXL1^WT^ patients (Fig. 2b). Unlike previously reported, we did observe heterogeneous gene expression profiles among the non-G646W *ASXL1* mutations^16^. The two ASXL1^MT^ patients with the most distally truncating mutations (Q695N and T957H) showed gene expression profiles intermediate between G646W and ASXL1^WT^ patients (Fig. 2b). We had a priori classified the samples based on the genotype (ASXL1^MT^ versus ASXL1^WT^). Both samples in question clustered with the other ASXL1^MT^ samples with regards to their epigenetic features despite the apparent differences in their gene expression profiles (Supplementary Fig. 2d). We therefore maintained our a priori sample classification based on the genotype (ASXL1^MT^ versus ASXL1^WT^) for all subsequent analyses. Functional annotation of the differentially expressed genes revealed several affected cellular processes including up-regulation of cell division (Supplementary Fig. 2e) and down-regulation of MHC class I dependent antigen presentation (Fig. 2c). Pathway analysis demonstrated an over-representation of genes involved in cell cycle (e.g. *CDK1*, *CCNA2*, *CCNB2*), DNA replication and repair (e.g. *MCM10*, *CDC6*, *CDC45*), gene expression (e.g. *CHEK1*, *RRM2*, *BRCA1*), signal transduction (e.g. *HLA-A*, *VCAM1*, *HLA-DQB1*), and antigen presentation (e.g. *CTSE*, *HLA-A*, *HLA-DQB1*) pathways (Supplementary Fig. 2f, Supplementary Data 5, 6).

### Truncating *ASXL1* mutations are associated with permissive promoter chromatin states supporting transcriptional up-regulation

**Fig. 3:**
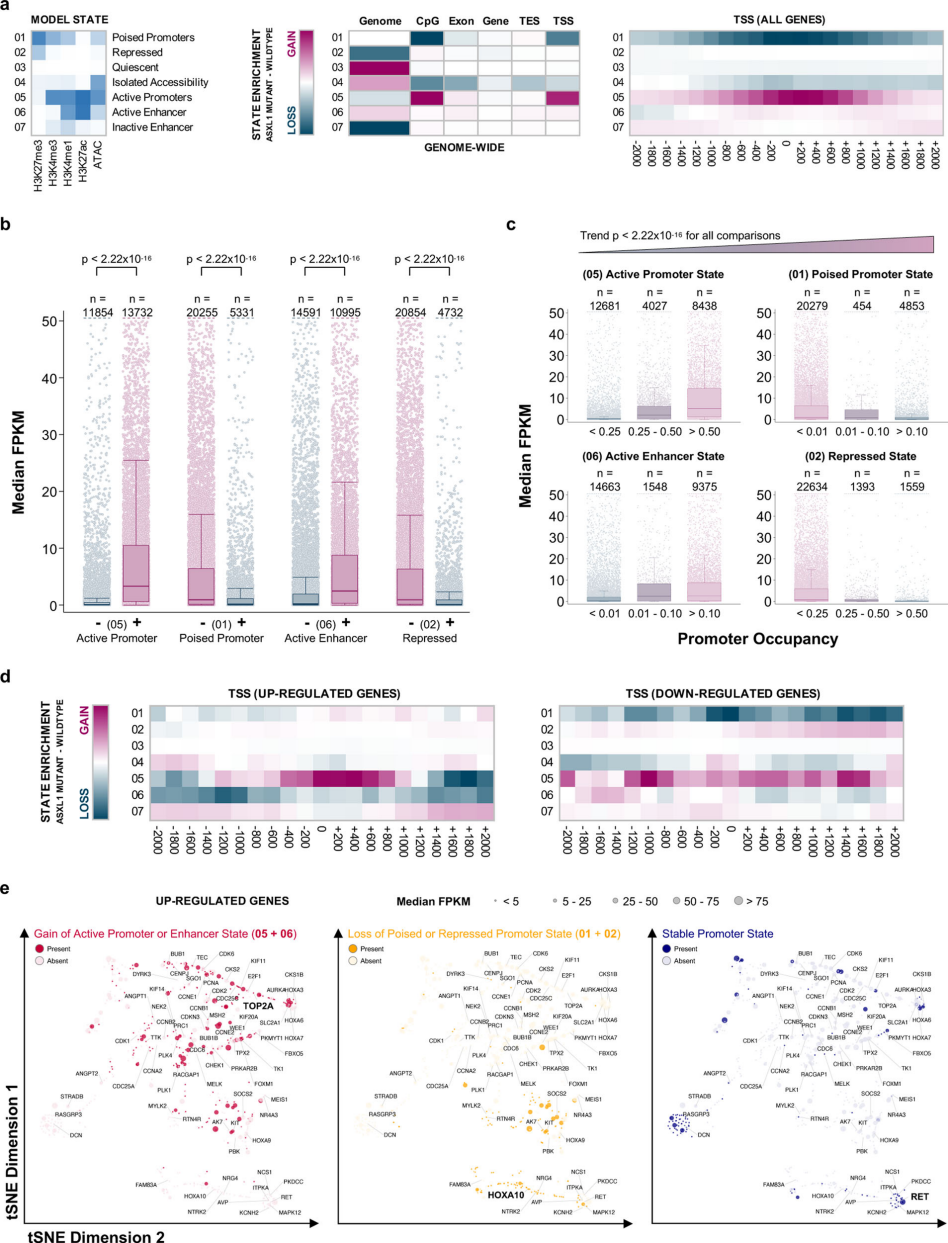
ASXL1^MT^ CMML is associated with permissive promoter chromatin states supporting transcriptional up-regulation. **a** Heatmaps showing the chromatin states discovered by hidden Markov modeling and the transition of chromatin states between ASXL1^WT^ and ASXL1^MT^ CMML. **b** Box and strip plots showing the association between the presence of a given chromatin promoter state and gene expression (transcriptome-wide) among patients with ASXL1^MT^ CMML (two-sided Mann–Whitney U test, raw *p*-values without adjustment for multiple hypothesis testing shown). **c** Box and strip plots showing the association between the extent of promoter occupancy of a given chromatin state and gene expression (transcriptome-wide) among patients with ASXL1^MT^ CMML (two-sided Cuzick’s test for trend, raw *p*-values without adjustment for multiple hypothesis testing shown). The associations shown in **b** and **c** validate the model’s ability to predict gene expression and suggest that the transitions of these chromatin states between ASXL1^WT^ and ASXL1^MT^ CMML may serve as a plausible explanation for the observed differences in gene expression. **d** Heatmaps showing the chromatin state transitions in promoter regions between ASXL1^WT^ and ASXL1^MT^ CMML for the up- (*n* = 707) and down-regulated (*n* = 122) genes separately. **e** Scatter plots showing the 707 up-regulated genes in two-dimensional tSNE space, clustered based on their promoter chromatin states. Color coding indicates the type of promoter chromatin state transition affecting each gene between ASXL1^WT^ and ASXL1^MT^ CMML. Marker size indicates the median gene expression among patients with ASXL1^MT^ CMML. The *HOXA* genes, *MEIS1*, and the mitotic kinases are labeled. One representative gene from each group of promoter chromatin state transitions is highlighted (bold print) and corresponding ChIP-seq signal tracks are shown in Supplementary Fig. 3e. Data are presented as standard Tukey boxplots (with the box encompassing Q1 to Q3, the median denoted as a central horizontal line in the box, and the whiskers covering the data within ±1.5 IQR in **3b** and **c**).

To understand the chromatin states associated with *ASXL1* mutations, we integrated data on histone modifications (H3K4me1, H3K4me3, H3K27ac, and H3K27me3 ChIP-seq) and chromatin accessibility (ATAC-seq) by fitting a 7-state hidden Markov model (Fig. 3a)^18^. First, we contrasted the two genotypes by subtracting the ASXL1^WT^ from the ASXL1^MT^ chromatin states. This genome-wide analysis revealed a transition of poised promoters and promoters with isolated chromatin accessibility to active promoters (loss of state 01 and 04, gain of state 05). This transition was mainly driven by H3K27ac and H3K4me1 gains in promoter regions. To verify that changes in promoter occupancy by the discovered chromatin states could serve as a plausible explanation for the observed transcriptional activity, we tested the associations between the presence of chromatin states and gene expression in ASXL1^MT^ CMML. As expected, the presence of active chromatin states was associated with increased gene expression (Fig. 3b). Conversely, the presence of poised and repressed chromatin states was associated with decreased gene expression. There was a dosage effect with greater promoter region occupancy of a given state being associated with a greater magnitude of this effect in the expected direction (Fig. 3c). Based on this model, promoter chromatin state transitions may serve as a plausible explanation for the observed changes in gene expression in ASXL1^MT^ CMML. Since the majority of the genes included in this genome-wide analysis were not expressed at all or not differentially expressed between the two genotypes, we performed a stratified analysis for the 707 up-regulated and 122 down-regulated genes in the ASXL1^MT^ patients identified by differential gene expression analysis (Fig. 3d). Among the down-regulated genes there were chromatin state transitions between ASXL1^MT^ and ASXL1^WT^ CMML involving a loss of poised chromatin (01) and chromatin with isolated accessibility (04) towards both active (05) and repressed states (02). Among the up-regulated genes there was a marked increase of the active promoter state (05) around the transcription start site and losses of active chromatin states (05, 06) in the flanking regions. We did observe a gain in active chromatin states and / or loss of poised and repressed chromatin states in the promoter regions of the majority of up-regulated genes including mitotic kinases and several *HOXA* cluster genes (Fig. 3e). However, for some genes (particularly two groups of relatively lowly expressed genes) we did not observe marked changes in promoter chromatin states between ASXL1^WT^ and ASXL1^MT^ CMML. Signal tracks for select up-regulated genes are shown in Supplementary Figure 3e. Importantly, some of the chromatin state transitions observed among the up-regulated genes (Fig. 3e) were not obvious when performing the genome-wide analysis (Fig. 3a). Consistent with previous observations, 5mC and 5hmC occupancy did not change significantly in the promoter regions of the differentially expressed genes (Supplementary Fig. 3a–d)^19^. These data support the notion that promoter chromatin state transitions between ASXL1^WT^ and ASXL1^MT^ CMML are a plausible explanation for the up-regulation of gene expression in ASXL1^MT^ CMML. On average, the transition from inactive to active promoter chromatin states is strongly associated with increased transcriptional activity, but considerable heterogeneity exists among the up-regulated genes.

### Transcriptional up-regulation in ASXL1^MT^ CMML is independent of gene body (hydroxy)methylation

**Fig. 4:**
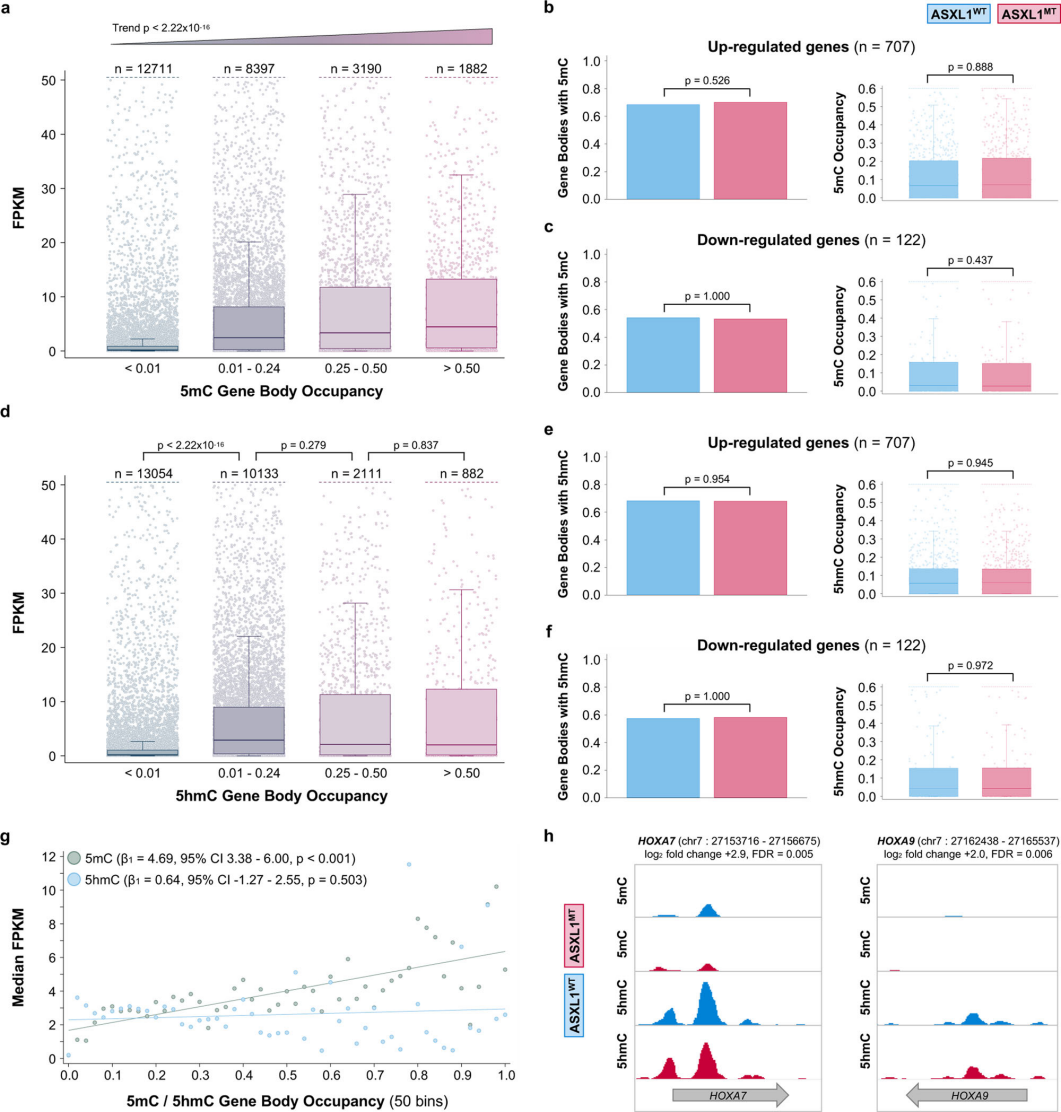
Gene body (hydroxy-)methylation is positively associated with gene expression but cannot serve as an explanation for the increased transcriptional activity given the lack of differential (hydroxy-)methylation between *ASXL1* genotypes. **a** Box and strip plots showing the association between the extent of gene body methylation and gene expression (transcriptome-wide) among patients with ASXL1^MT^ CMML (two-sided Cuzick’s test for trend, raw p-values without adjustment for multiple hypothesis testing shown). **b** Bar graphs showing the lack of differential gene body methylation between ASXL1^WT^ and ASXL1^MT^ CMML for the up-regulated genes. **c** Bar graphs showing the lack of differential gene body methylation between *ASXL1*^*WT*^ *and ASXL1*^*MT*^ CMML for the down-regulated genes. **d** Box and strip plots showing the association between the extent of gene body hydroxymethylation and gene expression (transcriptome-wide) among patients with ASXL1^MT^ CMML. **e** Bar graphs showing the lack of differential gene body hydroxymethylation between ASXL1^WT^ and ASXL1^MT^ CMML for the up-regulated genes. **f** Bar graphs showing the lack of differential gene body hydroxymethylation between ASXL1^WT^ and ASXL1^MT^ CMML for the down-regulated genes. **g** Scatter plot showing the association between the extent of gene body (hydroxy)methylation and gene expression among patients with ASXL1^MT^ CMML. Gene expression increases linearly with increases in gene body methylation. While the presence (compared to the absence) of gene body hydroxymethylation is strongly associated with increased gene expression, a greater extent of gene body hydroxymethylation is not associated with further increases in gene expression (threshold). The two-sided Wald test was used to test the model coefficients (raw p-values without adjustment for multiple hypothesis testing are shown). **h** Signal tracks showing the lack of differential (hydroxy-)methylation between ASXL1^WT^ and ASXL1^MT^ CMML for the up-regulated genes *HOXA7* and *HOXA9*. Data are presented as mean values (bars in **4b**, **c**, **e**, **f**) or standard Tukey boxplots (with the box encompassing Q1 to Q3, the median denoted as a central horizontal line in the box, and the whiskers covering the data within ±1.5 IQR in **4a**–**f**). The two-sided Mann–Whitney U test was used to compare groups in **4b**–**f**, raw *p*-values without adjustment for multiple hypothesis testing are shown.

Given this heterogeneity of promoter chromatin states across the up-regulated genes, we sought to interrogate potential alternative epigenetic regulatory mechanisms that may explain the observed transcriptional activity. Aberrant genome-wide DNA methylation is associated with adverse cytogenetics features, increased leukemic transformation rates, and inferior survival outcomes in CMML^20^. However, differential DNA methylation in CMML is known to predominantly affect hematopoiesis-specific enhancers rather than promoter regions and gene bodies^19,21^. Not having observed differences in (hydroxy)methylation of promoter regions of the up-regulated genes (Supplementary Fig. 3a–d), we interrogated gene body (hydroxy)methylation next. Gene body methylation was strongly associated with increased transcriptional activity (Fig. 4a). However, there was no differential gene body methylation between ASXL1^WT^ and ASXL1^MT^ CMML for either the up- (Fig. 4b) or down-regulated genes (Fig. 4c). Similarly, gene body hydroxymethylation was strongly associated with increased transcriptional activity (Fig. 4d). Again, there was no differential gene body hydroxymethylation between ASXL1^WT^ and ASXL1^MT^ CMML for either the up- (Fig. 4e) or down-regulated genes (Fig. 4f). In addition to the lack of differential (hydroxy)methylation in these stratified analyses, the co-mutations that might affect DNA (hydroxy)methylation were relatively balanced between the two *ASXL1* genotypes (Fig. 1c). To ensure that the presence of *TET2*, *DNMT3A*, or *IDH2* co-mutations did not confound the (hydroxy)methylation status, we performed a stratified analysis that demonstrated no significant difference in global DNA (hydroxy)methylation (Supplementary Figure 4a). We further validated these results using methylation microarrays (Supplementary Fig. 4b, c). While transcriptional activity increased both in the presence of gene body methylation and hydroxymethylation, the effects of the extent of methylation and hydroxymethylation on gene expression were different. While a greater extent of gene body methylation was associated with increased transcriptional activity (dosage effect), there was no such relationship for hydroxymethylation (threshold effect, Fig. 4g). Representative signal tracks for select genes of interest are shown in Fig. 4h. As an alternative unbiased approach, we identified 1595 differentially methylated regions (DMR, regions with FDR < 0.05) in the validation data set (microarray data). We mapped all hypermethylated regions to gene bodies requiring that there was no concurrent hypermethylation of the promoter region of the same gene. With this approach we identified one of the 707 up-regulated single genes (0.14%) with evidence of isolated gene body hypermethylation (*HBZ*, log_2_-fold change in gene expression 4.25, FDR = 0.0008, DMR area=1.61, DMR FDR = 0.018). These data support the notion that gene body (hydroxy)methylation is associated with increased gene expression. However, the lack of differential (hydroxy)methylation between ASXL1^WT^ and ASXL1^MT^ for the up-regulated genes make it an unlikely explanation for the observed increase in transcriptional activity.

### ASXL1^MT^-specific distal enhancers are independently associated with transcriptional activity

**Fig. 5:**
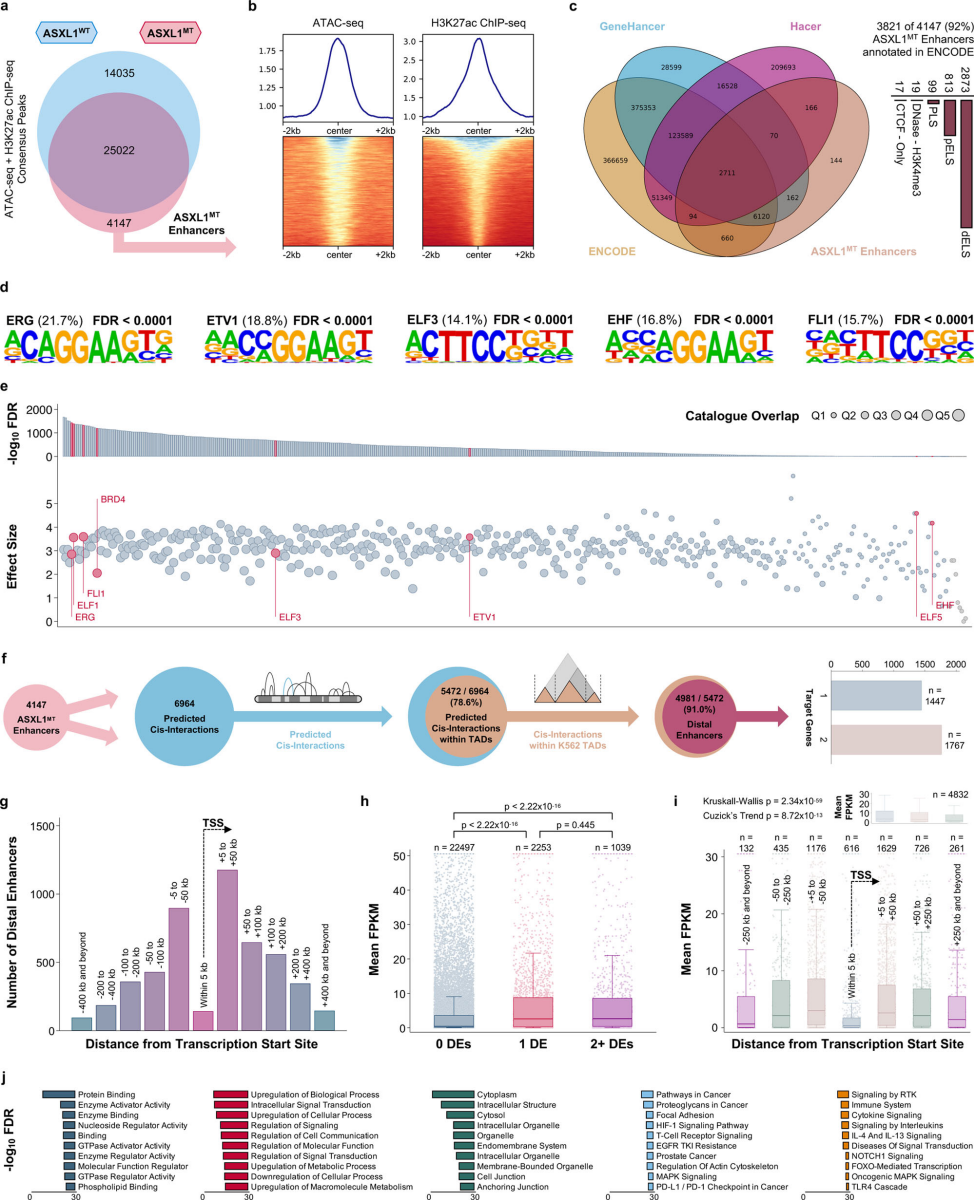
ASXL1^MT^-specific distal enhancers are positively associated with gene expression of their putative target genes and can serve as a plausible explanation for the increased transcriptional activity in ASXL1^MT^ CMML. **a** Venn diagram showing the co-mapping of chromatin accessibility and H3K27ac to identify ASXL1^MT^-specific cis-regulatory elements. **b** Signal curves and heatmaps showing the co-occurrence of DNA accessibility and H3K27ac in these ASXL1^MT^-specific cis-regulatory elements. **c** Venn diagram and bar graphs demonstrating the identity of these ASXL1^MT^-specific cis-regulatory elements (known enhancers, mostly distally located). **d** Position weight matrices generated from motif discovery show the over-representation of ETS transcription factors (top 5 enriched motifs) in the ASXL1^MT^-specific distal enhancers. **e** Validation of the predicted ETS transcription factor enrichment in the ASXL1^MT^-specific distal enhancers using publicly available human transcription factor ChIP-seq data. **f** Euler diagrams and bar graphs showing the association between the ASXL1^MT^-specific distal enhancers and putative target genes within leukemia-specific topologically associating domains. **g** Bar graphs showing the distribution of the ASXL1^MT^-specific distal enhancers by distance on the linear genome from the transcription start site of their putative target genes. **h** Box and strip plots demonstrating the association between the presence of distal enhancers and increased gene expression among patients with ASXL1^MT^ CMML (without evidence for a dosage effect of more than one distal enhancer). **i** Box and strip plots demonstrating the association between proximity of distal enhancer and putative target gene on the linear genome and increased gene expression of the putative target gene among patients with ASXL1^MT^ CMML. **j** Bar graphs showing the functional annotation of the putative target genes of the ASXL1^MT^-specific distal enhancers including receptor tyrosine kinase, cytokine, and oncogenic MAPK signaling. Data are presented as standard Tukey boxplots (with the box encompassing Q1 to Q3, the median denoted as a central horizontal line in the box, and the whiskers covering the data within ±1.5 IQR in **5h** and **i**).

Cis-regulatory elements are powerful regulators of transcription and frequently altered in myeloid neoplasms^22^. Enhancers have been associated with aberrant transcriptional activity and response to treatment in CMML^23^. We identified candidate cis-regulatory elements by integrating chromatin accessibility and histone acetylation data. To explain increased transcriptional activity among ASXL1^MT^ patients, we hypothesized that the responsible cis-regulatory elements would only be present in ASXL1^MT^ but not ASXL1^WT^ CMML (genotype-specific cis-regulatory elements). Regions with chromatin accessibility (ATAC-seq) which were concurrently marked by H3K27ac and unique to ASXL1^MT^ were considered candidate regions (Fig. 5a, b). There was no evidence for increased de-ubiquitination of H2AK119 in proximity of these genomic regions (Supplementary Fig. 5a). The vast majority of the identified regions (92%) were annotated in ENCODE, with 75% of these regions demonstrating a distal enhancer-like signature (Fig. 5c) and 2% being known super-enhancers^24–27^. Motif discovery revealed that these regions were predicted to bind ETS family transcription factors (Fig. 5d)^28^. Intersection with publicly available ChIP-seq data confirmed the presence of ETS family transcription factors in these ASXL1^MT^-specific enhancer regions (Fig. 5e)^29^. BRD4 was also among the top 20 enriched transcription factors (effect size 2.05, *p* < 2.22 × 10^−16^) and this enrichment was slightly more pronounced in the super-enhancer regions (effect size 2.59, *p* = 0.005). To associate the enhancers with putative target genes, we used a neighboring gene approach incorporating proximity on the linear genome, experimentally determined regulatory domains, and localization within conserved topologically associating domains (TADs)^30,31^. We excluded candidate regions overlapping with promoter regions and focused on distal enhancers (91% of the identified regions). These 3214 ASXL1^MT^-specific distal enhancers were implicated in 4981 cis-interactions with 3308 genes (Fig. 5f). Among these 3308 genes were 97 of the 707 up-regulated genes, an overlap unlikely to have occurred by chance (*p* = 2.22 × 10^−6^). Among these 97 up-regulated target genes were *MEIS1* and 17 mitotic kinases including *CDK1*, *CCNE1*, and *CDC20* (significant enrichment of mitotic cell cycle process, GO:1903047, FDR = 0.011). By virtue of the applied selection algorithm (GREAT) and additional constraints (TADs), most ASXL1^MT^-specific distal enhancers were localized between 5 and 200 kb of their associated target gene (Fig. 5g). The presence of a distal enhancer was positively associated with the expression of its target gene without evidence for a dosage effect when more than one enhancer was associated with a target gene (Fig. 5h). The effect of the presence of a distal enhancer on the expression of its target gene decreased with increasing distance on the linear genome (Fig. 5i). The genes associated with these ASXL1^MT^-specific distal enhancers were involved in oncogenic MAPK and receptor tyrosine kinase signaling (Fig. 5j). To understand if the presence of these enhancers is independently associated with increased gene expression, we fit multivariable-adjusted linear regression models. Among patients with ASXL1^MT^ CMML, the presence of these distal enhancers was independently associated with increased gene expression (Supplementary Fig. 5b). The magnitude of this effect (the presence of an ASXL1^MT^-specific distal enhancer) was comparable to a 20% increase in active promoter chromatin states or in gene body methylation. A measure of the relative importance of the different epigenetic regulatory mechanisms is shown in Supplementary Fig. 5c. Relevant regression diagnostics for the model are shown in Supplementary Fig. 5d–f. These data support the notion that ASXL1^MT^-specific distal enhancers are independently associated with increased oncogenic gene expression and their presence can serve as a plausible explanation for the observed increased transcriptional activity including the overexpression of mitotic kinases.

### ASXL1^MT^ CMML is characterized by increased intratumoral heterogeneity due to increased chromatin accessibility in transcription factor binding sites

**Fig. 6:**
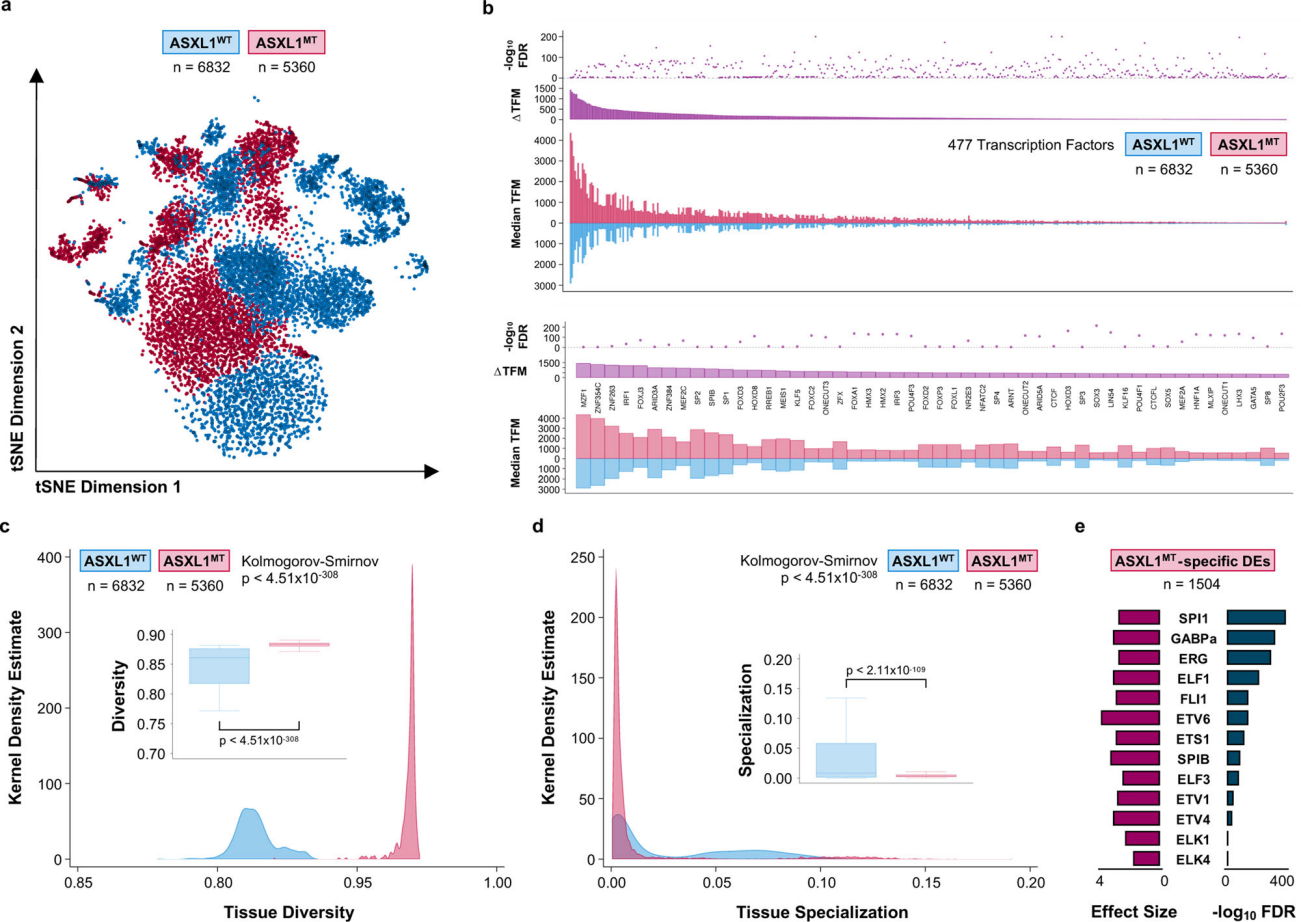
ASXL1^MT^ CMML is associated with increased intratumoral heterogeneity secondary to an extended repertoire of accessible distal enhancers. **a** Scatter plot showing 12192 single cells from CMML patients (stratified by *ASXL1* genotype) in two-dimensional tSNE space, clustered based on the accessibility of known transcription factor motifs. **b** Bar graphs and dot plot demonstrating the increased single-cell accessibility of binding sites for 476 transcription factors (top panel), ranked by the difference in accessible transcription factor motif (TFM) binding sites (scATAC-seq peaks with a given transcription factor motif) between ASXL1^WT^ and ASXL1^MT^ CMML. The top 50 transcription factors with increased accessible binding sites are magnified (bottom panel) and included key oncogenic myeloid transcription factors such as MZF1, MEF2C, and MEIS1. Source data are provided as a Source Data file. **c** Area graphs and box plots showing a measure of tissue diversity (based on single-cell entropies) for 12192 single cells from CMML patients (stratified by *ASXL1* genotype). The two-sample Kolmogorov-Smirnov test for equality of distribution functions was used to compare both distributions. Source data are provided as a Source Data file. **d** Area graphs and box plots showing a measure of tissue specialization (based on single-cell entropies) for 12192 single cells from CMML patients (stratified by *ASXL1* genotype). The two-sample Kolmogorov-Smirnov test for equality of distribution functions was used to compare both distributions (*p*-value). Source data are provided as a Source Data file. **e** Bar graphs showing the enrichment of ETS transcription factor motifs in 1504 ASXL1^MT^-specific distal enhancers identified by scATAC-seq (validation of bulk ATAC-seq findings). Data are presented as standard Tukey boxplots (with the box encompassing Q1 to Q3, the median denoted as a central horizontal line in the box, and the whiskers covering the data within ±1.5 IQR in **6c** and **d**).

Having identified ETS family transcription factors predicted to bind the ASXL1^MT^-specific distal enhancers in proximity to several of the up-regulated genes, we performed single-cell ATAC-seq on three patients with ASXL1^WT^ (6832 cells) and three patients with ASXL1^MT^ CMML (5360 cells) to validate our bulk ATAC-seq findings (Supplementary Fig. 6a–e). Cells from patients with ASXL1^MT^ CMML demonstrated increased chromatin accessibility as evidenced by an extended repertoire of accessible transcription factor motifs (Fig. 6b). Among the top motifs were key oncogenic myeloid transcription factors including MZF1 (known to stimulate proliferation and delay differentiation), MEF2C (linked to therapeutic resistance), and MEIS1 (synergistic with HOXA9 in inducing leukemogenesis)^32–34^. We hypothesized that the increased chromatin accessibility could drive intratumoral heterogeneity in ASXL1^MT^ CMML. We calculated measures of tissue diversity and specialization for both genotypes and observed an increase in diversity with a reciprocal decrease in specialization in ASXL1^MT^ CMML (Fig. 6c and d). When examining 1504 ASXL1^MT^-specific distal enhancers (identified by scATAC-seq), we again observed an enrichment of ETS family transcription factor motifs (Fig. 6e). Similarly, BRD4 was again among the top 5 enriched transcription factors in these regions (effect size 2.32, *p* < 2.22 × 10^−16^). These data support the notion that ASXL1^MT^ CMML is characterized by an increase in intratumoral heterogeneity with increased chromatin accessibility for transcription factors binding ASXL1^MT^-specific distal enhancers.

## Discussion

The gene expression profile of ASXL1^MT^ CMML is characterized by overexpression of proliferative genes, mirroring the clinical phenotype of leukocytosis, splenomegaly, resistance to epigenetic therapies (DNA methyltransferase inhibitors), and increased leukemic transformation rates^35^. Gaining a better understanding of the molecular mechanisms underlying this aberrant transcriptional activity is important for the development of novel therapies for patients with ASXL1^MT^ CMML. In this study we interrogated the epigenome of patients with ASXL1^MT^ CMML and observed a transition from poised and inactive to active chromatin states in promoter regions and increased chromatin accessibility exposing de novo distal enhancers correlating with the transcription of affected genes. We furthermore observed an increase in intratumoral heterogeneity due to an extended repertoire of transcription factor motifs, again involving ASXL1^MT^-specific distal enhancers. These results suggest an important role of oncogenic cis interactions for the sustained expression of key drivers of leukemogenesis in ASXL^MT^ CMML.

The three ASXL proteins (ASXL1, ASXL2 and ASXL3) are mammalian homologs of Addition of sex combs (Asx) in *Drosophila*, a protein that regulates the balance of trithorax (activating) and polycomb (repressive) functions. In myeloid neoplasms, the *ASXL1* gene is frequently affected by nonsense and frameshift mutations leading to truncation of the protein at the C-terminus and loss of the plant homeodomain (PHD)^15^. Hitherto, the associated proteins for the PHD of the Asx family remain unknown^36^. Investigations employing different disease models have shed light on the transcriptomic and epigenetic changes associated with ASXL1 loss and truncation. ASXL1 is thought to recruit chromatin modulators and transcription factors to alter transcriptional activity of genes involved in leukemogenesis. The exact molecular mechanisms associated with truncating *ASXL1* mutations however remain to be defined. Based on the interaction between ASXL1 and BAP1, a predominant loss of polycomb repressive complex 1 (PRC1)-mediated histone de-ubiquitination was the initial expected mechanism regulating transcriptional activity in ASXL1^MT^ patients^12,37^. However, observations in ASXL1^MT^ AML cell lines suggested a predominant loss of polycomb repressive complex 2 (PRC2)-mediated histone methylation (loss of H3K27me3) to be the regulatory mechanism at work and implicated the deregulation of posterior *HOXA* cluster genes as key factors in the ensuing leukemogenesis^9^. Observations from an ASXL1^MT^ murine model suggested a gain of function of PRC1-mediated effects rather than altered PRC2 activity (unaffected H3K27me3)^13^. The notion that increased de-ubiquitination is facilitating the transcriptional up-regulation through enhanced activity of the ASXL1-BAP1 complex was further confirmed in different ASXL1^MT^ cell lines, however, a decrease in H3K27me3 was observed at the same time in this disease model^11^. Loss of both H3K27me3 and H2AK119Ub promoting the expression of leukemogenic posterior *HOXA* genes has since been confirmed in cell lines and a murine model^10^. Another ASXL1^MT^ murine model revealed an increase in chromatin accessibility along with the recruitment of the bromodomain and extra-terminal domain family member BRD4, resulting in enhanced expression of genes involved in stem-cell maintenance and myeloid differentiation^14^. Overall, the sum of evidence from these mechanistic studies suggests that ASXL1 has a complex interactome, that truncating *ASXL1* mutations promote leukemogenesis by transcriptional up-regulation of leukemogenic drivers including posterior *HOXA* genes, and that these mutations recruit several effectors to alter the epigenome through histone modifications, increases in chromatin accessibility, and remodeling of enhancers^9–14^.

Given the complexity of human transcriptional regulation in vivo and the multitude of potential epigenetic mechanisms cooperating to regulate transcriptional activity, we interrogated the genome, transcriptome, and epigenome of ASXL1^MT^ CMML using primary patient samples. Employing conservative statistical methods and relying on a robust number of biological replicates, we observed significant changes in promoter chromatin states and distal enhancers in ASXL1^MT^ CMML. In general, these changes were less extreme than the ones observed in data generated from different hematopoietic precursor and leukemic cell lines^9,11^. We interpret these differences as a consequence of greater biological variability in primary patient samples and the statistical framework employed in this study. While previous studies have focused on global changes in histone modifications and considered them in isolation, we integrated several omics layers using an unbiased machine learning approach to capture the complexity of the epigenetic landscape to a greater extent. In doing so, we observed significant differences between global trends in chromatin remodeling and changes affecting the differentially expressed genes of interest. This may indicate that global assessments of chromatin remodeling are only part of the information required to develop effective targeted epigenetic therapies for specific leukemogenic mechanisms. For many of the up-regulated genes, we observed chromatin state transitions towards active states driven by H3K27ac deposition in promoter regions. Similarly, we observed a loss of H2K27me3 affecting the promoter regions of several of the up-regulated genes (either in isolation or in concert with the aforementioned gains in active chromatin states). For several up-regulated, yet relatively lowly expressed genes the model did not reveal marked state transitions in the promoter regions. The global increase in the active promoter state also included gains in H3K4me1, which is of particular interest since *KMT2A* fusions, partial tandem duplications, abnormalities of KMT2C/KMT2D/SET1D/2D (COMPASS family) are exceedingly rare in CMML^38^. Another open question concerns the locus-specific effects of truncating ASXL1 mutations. While we observed widespread remodeling of the epigenome, we do not fully understand how truncated ASXL1 exerts its effects selectively across the epigenome. This represents a limitation of the current study and future work in suitable disease models may help further refine the list of targets of truncated ASXL1. While ChIP-seq studies with antibodies directed against truncated ASXL1 may reveal genomic loci of direct interaction, other ASXL1-mutant-specific effects may be mediated through protein-protein interactions^39,40^. Furthermore, our gene expression data did not support the gene expression profiles observed in different murine myeloid disease models (e.g. lack of *HHEX* up- and lack of *SOX6* down-regulation in ASXL1^MT^ CMML) and it remains debatable how closely these models recapitulate human disease biology, given the inherent heterogeneity of human myeloid neoplasms^13,41^. In search of additional regulatory mechanisms for the up-regulated genes, we revisited promoter and gene body methylation without discovering compelling evidence for either mechanism being at work in ASXL1^MT^ CMML, despite the high prevalence of concurrent *TET2* mutations^35^. This is consistent with previous observations in CMML pointing towards the importance of hematopoiesis-specific enhancers and may further be explained by the relatively balanced distribution of co-mutations in this study^19,21^. In CMML, aberrant methylation is known to be associated with distinct clinical features such as high-risk karyotypes and therapeutic resistance^20,23^. At the same time, the most commonly employed treatment strategy for CMML is the inhibition of DNMT with DNA methyltransferase inhibitors (i.e. azacytidine ± cedazuridine, decitabine), which are thought to epigenetically restore normal hematopoiesis in a subset of patients without altering the mutational burden or inherent risk of leukemic transformation^42–44^. Furthermore, subsequent translational investigations in CD14-selected peripheral blood mononuclear cells demonstrated that differential promoter methylation does not correlate with transcription in CMML^45^. Our DNA immunoprecipitation and microarray methylation studies using unselected bone marrow mononuclear cells do confirm these findings, so do our RNA-seq results in CD34-selected, CD14-selected, and unselected bone marrow mononuclear cells. Extending the search for plausible additional regulatory mechanisms, we discovered a group of distal enhancers with interesting characteristics. First, these enhancers were specific for ASXL1^MT^ CMML. Second, these enhancers associated with several of the genes up-regulated in ASXL1^MT^ CMML. Third, these enhancers tightly correlated with transcription independent of the prevalent promoter chromatin states and gene body (hydroxy)methylation. A model including promoter chromatin states, ASXL1^MT^-specific distal enhancers, and gene body (hydroxy)methylation explained almost half of the variation in gene expression and adds to our understanding of the complexity of transcriptional regulation in ASXL1^MT^ CMML. These ASXL1^MT^-specific distal enhancers were highly enriched in ETS family transcription factor motifs as well as BRD4 and explained part of the observed increase in intratumoral heterogeneity at the single-cell level. The mapping of these enhancers and their association with key up-regulated leukemogenic drivers such as *MEIS1* and several mitotic kinases is of considerable interest given that the presence of such enhancers represents an explanation for the lineage- and context-specific transcriptional effects of novel therapeutic agents such as BET bromodomain inhibitors^46,47^. Observing BRD4 enrichment in the ASXL1^MT^-specific distal (super-)enhancers, further support the exploration of these novel therapeutics for individualized treatment approaches in ASXL1^MT^ CMML. Here we drew a detailed map of oncogenic distal enhancers unique to a high-risk phenotype of CMML, laying the foundation for future mechanistic studies defining the viability of these aberrant cis interactions to serve as therapeutic targets. Genotype-specific oncogenic chromatin interactions may be exploited for therapeutic benefit and serve as the rationale for early phase clinical trials with emerging epigenetic small molecule therapeutics in a disease that has not seen significant therapeutic advances in the last 25 years^48–51^.

## Methods

### Patient population, sample acquisition, and cell selection

To survey the (epi-)genetic landscape of human CMML, we obtained bone marrow mononuclear cells from 16 patients with WHO-defined CMML (Table 1), half of which had truncating *ASXL1* mutations. We performed targeted next generation sequencing of DNA, whole transcriptome RNA sequencing (RNA-seq), immunoprecipitation of DNA hydroxymethyl and methyl residues (DIP-seq), immunoprecipitation of the histone modifications H2AK119ub, H3K4me1, H3K4me3, H3K27ac, and H3K27me3 (ChIP-seq), and DNA transposase accessibility assays (ATAC-seq). The RNA-seq data were used to define the transcriptional activity in ASXL1^MT^ CMML and then correlated with known epigenetic regulatory mechanisms by integrating the ChIP-, DIP-, and ATAC-seq data. CMML is a malignant proliferation of monocytes and, unlike in acute myeloid leukemia, the fraction of CD34^+^ blasts is usually below 5%^35^. CD14 on the other hand is a marker of terminally differentiated monocytes as they appear in the peripheral blood and it is debatable whether the CD14^+^ cell population in the bone marrow represents the malignant cell population of interest in CMML. We refrained from further CD34- or CD14-sorting the bone marrow mononuclear cells for several reasons: First, the cell attrition rates due to CD34- and CD14-sorting would have made the multi-omics interrogation of primary patient samples for this study impossible (particularly the cell requirements for the ChIP-seq experiments). Second, we observed a tight correlation between the gene expression profiles of sorted and unsorted cells, raising the question whether cell sorting would have influenced the results significantly (Supplementary Fig. 2a–c). Third, our methylation analysis on unsorted cells confirms the results generated from CD14-sorted monocytes^45^. Avoiding excessive cell attrition due to sorting, we have been able to perform all analyses with at least five biological replicates per group. There were 16 patients included in this study and we performed experiments on all available samples for these patients. Supplementary Fig. 1c shows the samples that were included in the analysis after performing sample and data quality control. There were 14 samples for RNA-seq (87.5%), 15 for H2AK119ub ChIP-seq (93.8%), 15 for H3K27ac ChIP-seq (93.8%), 12 for H3K27me3 (75.0%), 14 for H3K4me1 ChIP-seq (87.5%), 11 for H3K4me3 ChIP-seq (68.8%), 15 for 5mC DIP-seq (93.8%), 15 for 5hmC DIP-seq (93.8%), and 11 for ATAC-seq (68.8%). The 16 patients included in this study were sampled from a population of 576 patients with WHO-defined CMML seen at the Mayo Clinic in Rochester, Minnesota^52^. All studies were approved by the Mayo Clinic Institutional Review Board. Written informed consent was obtained from all patients in accordance with the Declaration of Helsinki. Bone marrow mononuclear cells were collected in EDTA tubes and selected using Ficoll-Hypaque density gradient centrifugation (Sigma-Aldrich, St. Louis, United States). Genomic DNA was isolated using the ACCEL-NGS 1 S Plus DNA Library Kit (Swift Biosciences, Ann Arbor, United States). Total RNA was analyzed by preparing cDNA libraries generated in accord with the RNA-seq preparation protocol (Illumina, San Diego, United States). Viable bone marrow mononuclear cells were stored in freezing medium (10% DMSO, 40% fetal bovine serum, 40% RPMI) at −80 °C.

### DNA targeted next-generation sequencing

For 375 of the 576 patients complete clinical information as well as genetic information from a 36-gene panel targeted next-generation sequencing assay were available for prognostic modeling. The regions of these 36 genes were selected for custom target capture using Agilent SureSelect Target Enrichment Kit (Agilent Technologies, Santa Clara, United States). Libraries derived from each DNA sample were prepared using NEB Ultra II (New England Biolabs, Ipswitch, United States) and individually barcoded by dual indexing. Sequencing was performed on an HiSeq 4000 (Illumina) with 150 bp paired-end reads. Forty-eight pooled libraries per lane were sequenced to a median read depth of ~400x. The custom panel of target regions covered all coding regions and consensus splice sites from the following 36 genes: *ASXL1*, *CALR*, *CBL*, *CEBPA*, *DNMT3A*, *EZH2*, *FLT3*, *IDH1*, *IDH2*, *IKZF1*, *JAK2*, *KRAS*, *MPL*, *NPM1*, *NRAS*, *PHF6*, *PTPN11*, *RUNX1*, *SETBP1*, *SF3B1*, *SH2B3*, *SRSF2*, *TET2*, *TP53*, U*2AF1*, and *ZRSR2*. Paired-end reads were processed and analyzed as previously described^8^.

### RNA-seq and qPCR

RNA quality was determined using an Agilent Bioanalyzer RNA Nanochip or Caliper RNA assay. Library preparation was performed using TruSeq Stranded Total RNA (Illumina) and sequenced on an HiSeq 2500 (Illumina) with paired-end reads. RNA-seq sequencing reads were processed through the MAPRSeq bioinformatics workflow as previously described^53^. Reads were aligned to the GRCh38 reference genome and transcript counts calculated using featureCounts (v2.0.0)^54^. Differential expression was performed using DESeq2 (v1.30.1), clustering using heatmap.2 (v3.1.1, https://CRAN.R-project.org/package=gplots), and plotting using EnhancedVolcano (v1.8.0, https://github.com/kevinblighe/EnhancedVolcano)^55^. Pathway analyses were performed using gProfiler (v2021-05-01) and the Reactome Pathway Knowledgebase (v2021-5-7)^56,57^. Potential therapeutic targets among the up-regulated genes were identified by querying DGIdb (v3.0)^17^. For the validation of RNA-seq results we analyzed a subset of target genes by quantitative Reverse Transcription Polymerase Chain Reaction (RT-qPCR). Total RNA from the RNA-seq experiments was used to synthetize cDNA with the High-Capacity cDNA Reverse Transcription Kit (Applied Biosystems, Carlsbad, United States). A 1/3 dilution of the total cDNA was amplified by real-time PCR. Samples were prepared with PerfeCTa SYBR Green FastMix (Quanta BioSciences, Gaithersburg, United States), the primer sets are given in Supplementary Data 1.

### ChIP-seq

Approximately 100,000 cells from each sample were used for input for native chromatin immunoprecipitation (N-ChIP). Cells were lysed on ice for 20 min in lysis buffer containing 0.1% Triton X-100, 0.1% deoxycholate, and protease inhibitor. Extracted chromatin was digested with 90U of MNase enzyme (New England Biolabs) for 6 min at 25 °C. The reaction was quenched with 250 µM of EDTA post-digestion. A mix of 1.0% Triton X-100 and 1.0% deoxycholate was added to the digested samples and incubated on ice for 20 min. Digested chromatin was pooled and pre-cleared in IP buffer (20 mM Tris-HCl [pH 7.5], 2 mM EDTA, 150 mM NaCl, 0.1% Triton X-100, and 0.1% deoxycholate) plus protease inhibitors with pre-washed Protein A/G Dynabeads (Thermo Fisher Scientific, Waltham, United States) at 4 °C for 1.5 h. Supernatants were removed from the beads and transferred to a 96-well plate containing the antibody-bead complex. Following an overnight 4 °C incubation, samples were washed twice with low salt buffer (20 mM Tris-HCl [pH 8.0], 0.1% SDS, 1.0% Triton X-100, 2 mM EDTA, and 150 mM NaCl) and twice with high salt buffer (20 mM Tris-HCl [pH 8.0], 0.1% SDS, 1.0% Triton X-100, 2 mM EDTA, and 500 mM NaCl). DNA-antibody complexes were eluted in elution buffer (100 mM NaHCO_3_, 1.0% SDS), incubated at 65 °C for 90 min. Protein digestion was performed on the eluted DNA samples at 50 °C for 30 min using protease mix (QIAGEN, Venlo, Netherlands). ChIP DNA was purified using Sera-Mag beads (Thermo Fisher Scientific) with 30% PEG before library construction. H2AK119ub and H3K27ac ChIP-seq was performed as previously described^58^. The antibodies were used at a dilution of 1:250 (H3K27me3), 1:500 (H3K4me1 and H3K4me3), and 1:1000 (H2AK119ub and H3K27ac). Libraries were prepared by following a modified Illumina paired-end protocol and sequenced on an HiSeq 2500 (Illumina) to a median depth of ~25 million (H3K4me1 and H3K4me3) or ~50 million reads (H3K27me3 and Input). Reads were aligned to the GRCh38 reference genome using bowtie2 (v2.3.3.1)^59^.

### DIP-seq and methylation microarrays

Genomic DNA was isolated and submitted to the Mayo Clinic Epigenomics Development Laboratory (EDL) for DNA immunoprecipitation and library preparation. The 5mC-33D3 (C15200081) monoclonal antibody (Diagenode, Denville, United States), an in-house developed 5hmC antibody (EDL), and the 53017 bridging antibody (Active Motif, Carlsbad, United States) were used. The antibodies were used at a dilution of 1:110 (5hmC) and 1:340 (5mC). Libraries were prepared by following a modified Illumina paired-end protocol and sequenced on a HiSeq 4000 (Illumina). Reads were aligned to the GRCh38 reference genome using the Burrows-Wheeler Aligner^60^. For the validation of DIP-seq results we analyzed a subset of samples on the Infinium MethylationEPIC (850 K) array (Illumina) according to the manufacturer’s specifications. Signal intensities were processed and normalized using Minfi (v1.36.0) using subset within-array quantile normalization^61,62^. CpGs below the detection threshold (*p* < 0.010) as well as unreliable and cross-reactive probes were removed, leaving 787403 CpGs for downstream analyses. Differentially methylated regions were identified using bumphunter (v1.32.0)^63^.

### ATAC-seq and single-cell ATAC-seq

DNA for ATAC-seq was prepared from 50,000 cells following the OMNI-ATAC procedures as described by *Corces* et al. with modifications using the Nextera kit (Illumina)^64^. The cells were lysed for 3 min on ice and transposed for 30 min at 37 °C following clean-up. The DNA libraries were prepared with 5–10 cycles of PCR amplification with the NEB High Fidelity Master Mix (New England Biolabs, Ipswich, United States). Clean-up was done using the Zymo DNA Clean and Concentrator kit (Zymo Research, Irvine, United States) and followed with AMPure XP (Beckman Coulter, Brea, United States) bead clean-up to remove primer dimers and under-digested chromatin. Sequencing was performed on an HiSeq 4000 (Illumina) to a depth of ~30 million reads per sample. Reads were aligned to GRCh38 using bowtie2^59^. For the single-cell chromatin transposase accessibility assays (scATAC-seq) cryopreserved bone marrow mononuclear cells were thawed and resuspended following an established workflow (thawing, resuspension, sequential dilution, centrifugation, straining, viability assessment) and approximately 100000 viable mononuclear cells per sample were subjected to transposase assays (exposing buffered nuclei to Tn5 transposase) before proceeding to single-cell partitioning into gel beads in emulsion, barcoding, library construction, and sequencing following established 10X Genomics protocols. The target cell recovery was approximately 2000 cells per sample. For details on the 10X Genomics Chromium platform including demonstrated protocols on sample preparation, library construction, instrument settings, and sequencing parameters please see the manufacturer’s resources (https://support.10xgenomics.com/single-cell-atac). Genomic libraries were sequenced on an HiSeq 4000 (Illumina) before demultiplexing, alignment to the reference genome, and post-alignment quality control. The 10X Genomics Cell Ranger ATAC software (v2.0) was used for demultiplexing, alignment of the reads to the GRCh38 reference genome, filtering and quality control, counting of barcodes and unique molecular identifiers, identification of transposase cut sites, detection of accessible chromatin peaks, count matrix generation for peaks and transcription factors.

### Consensus peak calling

Aligned reads from the different immunoprecipitation and accessibility sequencing experiments were sorted and indexed using samtools (v1.9) and peaks were called using MACS2 (v3.0.0a6) with input controls (except ATAC-seq)^65,66^. Peak calling with default parameters was performed before subjecting the peaks to the MSPC (v5.4.0) consensus peak calling algorithm^67^. By convention H3K4me1, H2AK119ub, and H3K27me3 peaks were called as “broad peaks” and the remaining marks were called as “narrow peaks”. To leverage the power of several biological replicates per analysis we employed a standard peak calling threshold in MACS2 (FDR < 0.050) before applying a more stringent threshold in the subsequent MSPC step (weak threshold *p* < 1.00 × 10^−4^, stringency threshold *p* < 1.00 × 10^−8^). The UCSC Genome Browser and deepTools (v3.5.0) were used for signal visualization^68,69^. For visualization purposes averaged, input-corrected average signal tracks were created (except ATAC-seq where no input was used) using wiggletools mean (v1.2) and deepTools bamCoverage / bigwigCompare^70^.

### Data analysis

Data are presented as median (range) unless denoted otherwise. Medians were the preferred measure of central tendency and non-parametric hypothesis tests were used for comparisons unless stated otherwise. Continuous variables were compared using the Mann-Whitney-U test, categorical variable using Fischer’s exact test. Trends across ordered groups were assessed using Cuzick’s test for trend. The equality of single-cell entropy distributions was evaluated using the two-sample Kolmogorov-Smirnov test. Overall and leukemia-free survival estimates were calculated using the Kaplan and Meier method^71^. Overall survival was defined as the time from diagnosis to death and patients who were alive at the end of follow-up were censored. Leukemia-free survival was defined as the time from diagnosis to leukemic transformation or death and patients who were alive and free of acute leukemia at the end of follow-up were censored. The log-rank test was used to compare time to event data in subgroups. Multivariable-adjusted (Cox) proportional hazards regression models were used to assess the association between clinical and genetic parameters of interest and overall survival^72^. Violations of the proportional hazards assumption were evaluated using Schoenfeld residuals. Hidden Markov modeling (ChromHMM v1.22) was used to discover and characterize the presence of chromatin states in promoter regions (TSS ± 2000bp)^18^. We fit 5- to 15-state models and judged their goodness of fit by each model’s ability to discriminate important chromatin states (active promoter, poised promoter, active enhancer, inactive enhancer, repressed, quiescent) without creating an excessive number of combinatorial states within each epigenetic mark. Based on the ability to sufficiently discriminate the activity of promoters and enhancers, the 7-state model was felt to be the most parsimonious model with acceptable fit for the data. Candidate cis-regulatory elements were validated using the ENCODE, GeneHancer, and Hacer databases^24,25,27^. To annotate and associate these regions with potential target genes, the Genomic Regions Enrichment of Annotations Tool (GREAT v4.0.4) was used^30^. To increase the specificity of the predicted (enhancer-promoter) cis-interactions, we included only those that did not violate the boundaries of topologically associating domains established in K562 cells^31^. Super-enhancers were identified by intersecting the candidate cis-regulatory elements with known super-enhancers in K562 cells^26^. De novo motif analysis was performed using Hypergeometric Optimization of Motif EnRichment (HOMER v3.0)^28^. Candidate transcription factors predicted to bind these regions were then validated by querying the ReMap 2020 database^29^. To estimate the independent effect of each epigenetic regulatory mechanism on gene expression, we employed multivariable-adjusted linear regression. Given the heavily left-skewed distribution and overdispersion of transcript count data we performed a log-transformed of the transcript count data (adding a pseudocount). We then used ordinary least squares regression to model the transformed transcript count data and exponentiated the obtained regression coefficients for ease of interpretation of the effect sizes (*e*^β^ − 1). The model included the active and inactive chromatin states, gene body (hydroxy)methylation, and the presence of an associated distal enhancer as parameters. We used the *lmg* metric (hierarchical partitioning of R^2^) as an estimate of relative importance for each model paramaters^73,74^. Routine regression diagnostics were employed to evaluate model assumptions and model fit (including assessments of influence, leverage, and multicollinearity). Observations with excessive leverage (*h* > [(2 * *k*)/*n*]) and influence (Cook’s D > [4/(*n* − *k* − 1)]) were removed (*n* denoting the sample size and *k* the number of model parameters). Quality control, integration, normalization (TF-IDF), scaling, feature selection, clustering, and dimensionality reduction (SVD) of the scATAC-seq data was performed using Seurat (v4.0.5)^75^. Batch correction was performed using Harmony (v0.1.0)^76^. Single-cell entropies were calculated using BioQC (v1.18.0)^77,78^.

### Reporting summary

Further information on research design is available in the Nature Research Reporting Summary linked to this article.

## Supplementary information

Supplementary Information

Description of Additional Supplementary Files

Supplementary Data 1

Supplementary Data 2

Supplementary Data 3

Supplementary Data 4

Supplementary Data 5

Supplementary Data 6

Reporting Summary

## Data Availability

The raw RNA sequencing data generated in this study have been deposited in the Gene Expression Omnibus (GEO) database under accession code GSE159543. The raw ChIP, DIP, and ATAC sequencing data generated in this study have been deposited in the GEO database under accession code GSE159886. The publicly available transcription factor ChIP sequencing data used in this study are available in the ReMap 2020 database [https://remap2020.univ-amu.fr]. The publicly available data on candidate cis-regulatory elements used in this study are available through the ENCODE database [https://www.encodeproject.org], the GeneHancer tracks in the UCSC Genome Browser [https://genome.ucsc.edu], and the Hacer database [http://bioinfo.vanderbilt.edu/AE/HACER/]. Source data are provided with this paper for Fig. 1d and Fig. 6b-d. The remaining data are available within the Article, Supplementary Information, or Source Data files. Source data are provided with this paper.

## Source data

Source Data

## References

[1] Metzeler KH (2016). Spectrum and prognostic relevance of driver gene mutations in acute myeloid leukemia. Blood.

[2] Papaemmanuil E (2016). Genomic classification and prognosis in acute myeloid leukemia. N. Engl. J. Med..

[3] Patnaik, M. M. & Tefferi, A. Chronic myelomonocytic leukemia: 2020 update on diagnosis, risk stratification and management. *Am. J. Hematol.* **95**, 97–115 (2020). 10.1002/ajh.25684 31736132

[4] Gelsi-Boyer V (2010). ASXL1 mutation is associated with poor prognosis and acute transformation in chronic myelomonocytic leukaemia. Br. J. Haematol..

[5] Itzykson R (2013). Prognostic score including gene mutations in chronic myelomonocytic leukemia. J. Clin. Oncol..

[6] Idossa D (2018). Mutations and karyotype predict treatment response in myelodysplastic syndromes. Am. J. Hematol..

[7] Elena C (2016). Integrating clinical features and genetic lesions in the risk assessment of patients with chronic myelomonocytic leukemia. Blood.

[8] Patnaik MM (2014). ASXL1 and SETBP1 mutations and their prognostic contribution in chronic myelomonocytic leukemia: a two-center study of 466 patients. Leukemia.

[9] Abdel-Wahab O (2012). ASXL1 mutations promote myeloid transformation through loss of PRC2-mediated gene repression. Cancer Cell.

[10] Asada S (2018). Mutant ASXL1 cooperates with BAP1 to promote myeloid leukaemogenesis. Nat. Commun..

[11] Balasubramani A (2015). Cancer-associated ASXL1 mutations may act as gain-of-function mutations of the ASXL1-BAP1 complex. Nat. Commun..

[12] Campagne A (2019). BAP1 complex promotes transcription by opposing PRC1-mediated H2A ubiquitylation. Nat. Commun..

[13] Nagase R (2018). Expression of mutant Asxl1 perturbs hematopoiesis and promotes susceptibility to leukemic transformation. J. Exp. Med..

[14] Yang H (2018). Gain of function of ASXL1 truncating protein in the pathogenesis of myeloid malignancies. Blood.

[15] Gelsi-Boyer V (2009). Mutations of polycomb-associated gene ASXL1 in myelodysplastic syndromes and chronic myelomonocytic leukaemia. Br. J. Haematol..

[16] Metzeler KH (2011). ASXL1 mutations identify a high-risk subgroup of older patients with primary cytogenetically normal AML within the ELN Favorable genetic category. Blood.

[17] Cotto KC (2018). DGIdb 3.0: a redesign and expansion of the drug-gene interaction database. Nucleic Acids Res..

[18] Ernst J, Kellis M (2012). ChromHMM: automating chromatin-state discovery and characterization. Nat. Methods.

[19] Yamazaki J (2012). Effects of TET2 mutations on DNA methylation in chronic myelomonocytic leukemia. Epigenetics.

[20] Palomo L (2018). DNA methylation profile in chronic myelomonocytic leukemia associates with distinct clinical, biological and genetic features. Epigenetics.

[21] Yamazaki J (2015). TET2 mutations affect non-CpG island DNA methylation at enhancers and transcription factor-binding sites in chronic myelomonocytic leukemia. Cancer Res..

[22] Bhagwat AS, Lu B, Vakoc CR (2018). Enhancer dysfunction in leukemia. Blood.

[23] Meldi K (2015). Specific molecular signatures predict decitabine response in chronic myelomonocytic leukemia. J. Clin. Invest.

[24] Encode Project Consortium. (2012). An integrated encyclopedia of DNA elements in the human genome. Nature.

[25] Fishilevich S (2017). GeneHancer: genome-wide integration of enhancers and target genes in GeneCards. Database.

[26] Khan A, Zhang X (2016). dbSUPER: a database of super-enhancers in mouse and human genome. Nucleic Acids Res..

[27] Wang J (2019). HACER: an atlas of human active enhancers to interpret regulatory variants. Nucleic Acids Res..

[28] Heinz S (2010). Simple combinations of lineage-determining transcription factors prime cis-regulatory elements required for macrophage and B cell identities. Mol. Cell.

[29] Cheneby J (2020). ReMap 2020: a database of regulatory regions from an integrative analysis of Human and Arabidopsis DNA-binding sequencing experiments. Nucleic Acids Res..

[30] McLean CY (2010). GREAT improves functional interpretation of cis-regulatory regions. Nat. Biotechnol..

[31] Rao SS (2014). A 3D map of the human genome at kilobase resolution reveals principles of chromatin looping. Cell.

[32] Brown FC (2018). MEF2C phosphorylation is required for chemotherapy resistance in acute myeloid leukemia. Cancer Disco..

[33] Collins C (2014). C/EBPalpha is an essential collaborator in Hoxa9/Meis1-mediated leukemogenesis. Proc. Natl Acad. Sci. USA.

[34] Robertson KA (1998). The myeloid zinc finger gene (MZF-1) delays retinoic acid-induced apoptosis and differentiation in myeloid leukemia cells. Leukemia.

[35] Coltro G (2020). Clinical, molecular, and prognostic correlates of number, type, and functional localization of TET2 mutations in chronic myelomonocytic leukemia (CMML)-a study of 1084 patients. Leukemia.

[36] Peng H (2018). Familial and somatic BAP1 mutations inactivate ASXL1/2-mediated allosteric regulation of BAP1 deubiquitinase by targeting multiple independent domains. Cancer Res..

[37] Scheuermann JC (2010). Histone H2A deubiquitinase activity of the polycomb repressive complex PR-DUB. Nature.

[38] Patnaik MM (2018). Therapy related-chronic myelomonocytic leukemia (CMML): molecular, cytogenetic, and clinical distinctions from de novo CMML. Am. J. Hematol..

[39] Yamamoto K (2021). A histone modifier, ASXL1, interacts with NONO and is involved in paraspeckle formation in hematopoietic cells. Cell Rep..

[40] Zhang P (2018). Loss of ASXL1 in the bone marrow niche dysregulates hematopoietic stem and progenitor cell fates. Cell Disco..

[41] Takeda R (2020). HHEX promotes myeloid transformation in cooperation with mutant ASXL1. Blood.

[42] Coston T (2019). Suboptimal response rates to hypomethylating agent therapy in chronic myelomonocytic leukemia; a single institutional study of 121 patients. Am. J. Hematol..

[43] Fenaux P (2009). Efficacy of azacitidine compared with that of conventional care regimens in the treatment of higher-risk myelodysplastic syndromes: a randomised, open-label, phase III study. Lancet Oncol..

[44] Merlevede J (2016). Mutation allele burden remains unchanged in chronic myelomonocytic leukaemia responding to hypomethylating agents. Nat. Commun..

[45] Franzini A (2019). The transcriptome of CMML monocytes is highly inflammatory and reflects leukemia-specific and age-related alterations. Blood Adv..

[46] Letson C (2019). Bromodomain and extra terminal domain (BET) inhibitors sensitize chronic myelomonocytic leukemia (CMML) to PIM inhibition via downregulation of Mir-33a. Blood.

[47] Roe JS, Mercan F, Rivera K, Pappin DJ, Vakoc CR (2015). BET bromodomain inhibition suppresses the function of hematopoietic transcription factors in acute myeloid leukemia. Mol. Cell.

[48] Bushweller JH (2019). Targeting transcription factors in cancer - from undruggable to reality. Nat. Rev. Cancer.

[49] Chen A, Koehler AN (2020). Transcription factor inhibition: lessons learned and emerging targets. Trends Mol. Med..

[50] Itzykson R (2020). Decitabine versus hydroxyurea for advanced proliferative CMML: results of the Emsco randomized phase 3 Dacota trial. Blood.

[51] Wattel E (1996). A randomized trial of hydroxyurea versus VP16 in adult chronic myelomonocytic leukemia. Groupe Francais des Myelodysplasies and European CMML Group. Blood.

[52] Arber DA (2016). The 2016 revision to the World Health Organization classification of myeloid neoplasms and acute leukemia. Blood.

[53] Kalari KR (2014). MAP-RSeq: Mayo analysis pipeline for RNA sequencing. BMC Bioinforma..

[54] Liao Y, Smyth GK, Shi W (2014). featureCounts: an efficient general purpose program for assigning sequence reads to genomic features. Bioinformatics.

[55] Love MI, Huber W, Anders S (2014). Moderated estimation of fold change and dispersion for RNA-seq data with DESeq2. Genome Biol..

[56] Jassal B (2020). The reactome pathway knowledgebase. Nucleic Acids Res..

[57] Raudvere U (2019). g:Profiler: a web server for functional enrichment analysis and conversions of gene lists (2019 update). Nucleic Acids Res..

[58] Zhong J (2017). Purification of nanogram-range immunoprecipitated DNA in ChIP-seq application. BMC Genomics.

[59] Langmead B, Salzberg SL (2012). Fast gapped-read alignment with Bowtie 2. Nat. Methods.

[60] Li H, Durbin R (2009). Fast and accurate short read alignment with Burrows-Wheeler transform. Bioinformatics.

[61] Aryee MJ (2014). Minfi: a flexible and comprehensive Bioconductor package for the analysis of Infinium DNA methylation microarrays. Bioinformatics.

[62] Maksimovic J, Gordon L, Oshlack A (2012). SWAN: Subset-quantile within array normalization for illumina infinium HumanMethylation450 BeadChips. Genome Biol..

[63] Jaffe AE (2012). Bump hunting to identify differentially methylated regions in epigenetic epidemiology studies. Int J. Epidemiol..

[64] Corces MR (2018). The chromatin accessibility landscape of primary human cancers. Science.

[65] Li H (2009). The sequence alignment/Map format and SAMtools. Bioinformatics.

[66] Zhang Y (2008). Model-based analysis of ChIP-Seq (MACS). Genome Biol..

[67] Jalili V, Matteucci M, Masseroli M, Morelli MJ (2015). Using combined evidence from replicates to evaluate ChIP-seq peaks. Bioinformatics.

[68] Kent WJ (2002). The human genome browser at UCSC. Genome Res..

[69] Ramirez F (2016). deepTools2: a next generation web server for deep-sequencing data analysis. Nucleic Acids Res..

[70] Zerbino DR, Johnson N, Juettemann T, Wilder SP, Flicek P (2014). WiggleTools: parallel processing of large collections of genome-wide datasets for visualization and statistical analysis. Bioinformatics.

[71] Kaplan EL, Meier P (1958). Nonparametric estimation from incomplete observations. J. Am. Stat. Assoc..

[72] Cox DR (1972). Regression Models and Life-Tables. J. R. Stat. Soc.: Ser. B.

[73] Chevan A, Sutherland M (1991). Hierarchical partitioning. Am. Statistician.

[74] Groemping U (2006). Relative importance for linear regression in R: the package relaimpo. J. Stat. Softw..

[75] Stuart T (2019). Comprehensive Integration of Single-. Cell Data. Cell.

[76] Korsunsky I (2019). Fast, sensitive and accurate integration of single-cell data with Harmony. Nat. Methods.

[77] Martinez O, Reyes-Valdes MH (2008). Defining diversity, specialization, and gene specificity in transcriptomes through information theory. Proc. Natl Acad. Sci. USA.

[78] Zhang JD (2017). Detect tissue heterogeneity in gene expression data with BioQC. BMC Genomics.

