# Supplementary material for: Oncogenic gene expression and epigenetic remodeling of cis-regulatory elements in ASXL1-mutant chronic myelomonocytic leukemia
Source: Nat Commun. 2022 Mar 17;13:1434. doi: 10.1038/s41467-022-29142-6 (PMC8931048; doi:10.1038/s41467-022-29142-6)
Supplement: Supplementary file 1 — Supplementary Information [file 41467_2022_29142_MOESM1_ESM.pdf]

## SUPPLEMENTARY INFORMATION

# Oncogenic Gene Expression in ASXL1-mutant Chronic Myelomonocytic Leukemia is Associated with Epigenetic Remodeling of Promoters and Enhancers


Moritz Binder[1,2], Ryan M. Carr[1], Terra L. Lasho[1], Christy M. Finke[1], Abhishek A. Mangaonkar[1], Christopher L. Pin[3], Kurt R. Berger[3], Amelia Mazzone[2,4], Sandeep Potluri[5], Tamas Ordog[2], Keith D. Robertson[2], David L. Marks[6], Martin E. Fernandez-Zapico[6], Alexandre Gaspar-Maia[2,4*], and Mrinal M. Patnaik[1,2*]

[1] Division of Hematology, Mayo Clinic, Rochester, MN, United States

[2] Epigenomics Program, Center for Individualized Medicine, Mayo Clinic, Rochester, MN, United States

[3] Lawson Health Research Institute, University of Western Ontario, London, ON, Canada

[4] Department of Laboratory Medicine and Pathology, Mayo Clinic, Rochester, MN, United States

[5] Institute of Cancer and Genomic Sciences, University of Birmingham, Birmingham, United Kingdom

[6] Schulze Center for Novel Therapeutics, Division of Oncology Research, Mayo Clinic, Rochester, MN, United States

Correspondence to:

Mrinal M. Patnaik, MD
Division of Hematology

Associate Professor of Medicine
Mayo Clinic College of Medicine and Science

Mayo Clinic
200 1st Street SW, MN 55905
United States

Alexandre Gaspar-Maia, PhD
Department of Laboratory Medicine and Pathology

Assistant Professor of Laboratory Medicine and Pathology
Mayo Clinic College of Medicine and Science


## SUPPLEMENTARY INFORMATION


1. Supplementary Figure 1 (page 2-3)
2. Supplementary Figure 2 (page 4-5)
3. Supplementary Figure 3 (page 6-7)
4. Supplementary Figure 4 (page 8-9)
5. Supplementary Figure 5 (page 10-11)
6. Supplementary Figure 6 (page 12-13)

# SUPPLEMENTARY FIGURE 1

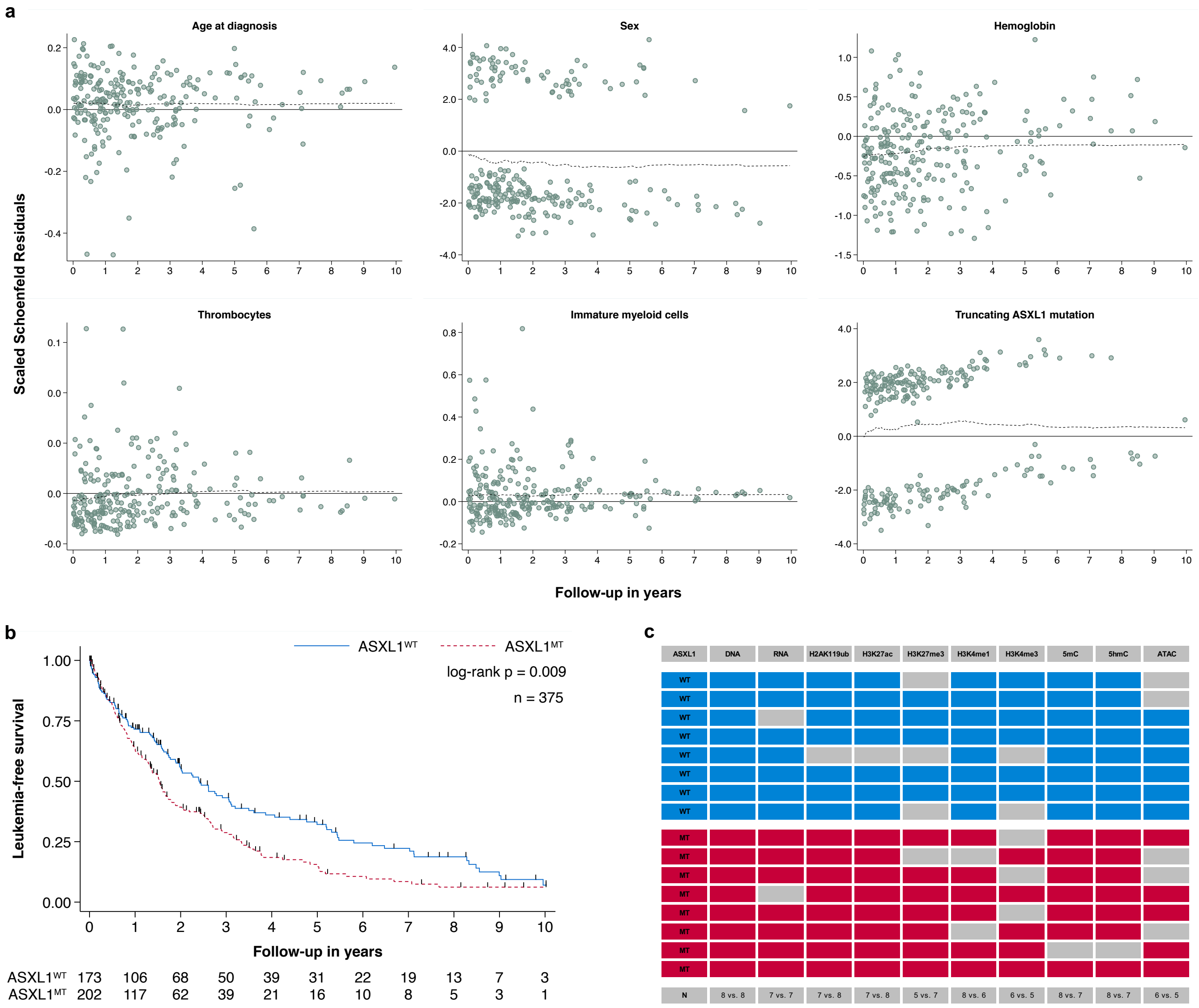

**Supplementary Figure 1** showing additional information on survival analysis and sample selection. **a**, Scatter plots showing the scaled Schoenfeld residuals for all parameters included in the multivariable-adjusted proportional hazards regression model (regression diagnostics). There were no obvious violations of the proportional hazard assumption for any of the prognostic factors included in the multivariable-adjusted regression model used to demonstrate the independent prognostic impact of truncating *ASXL1* mutations on overall survival. **b**, Kaplan-Meier plot showing the association between truncating *ASXL1* mutations and leukemia-free survival. This relationship was similar to the association observed with overall survival given the relatively low number of leukemic transformation events. **c**, Heatmap of patient samples showing the number of biological replicates for each omics layer ($ASXL1^{WT}$ in blue, $ASXL1^{MT}$ in red). Samples were excluded (gray) either because of quality control measures or lack of sufficient bone marrow mononuclear cells for additional experiments. The number of biological replicates for each omics data type was at least five samples per group for all analyses.

# SUPPLEMENTARY FIGURE 2

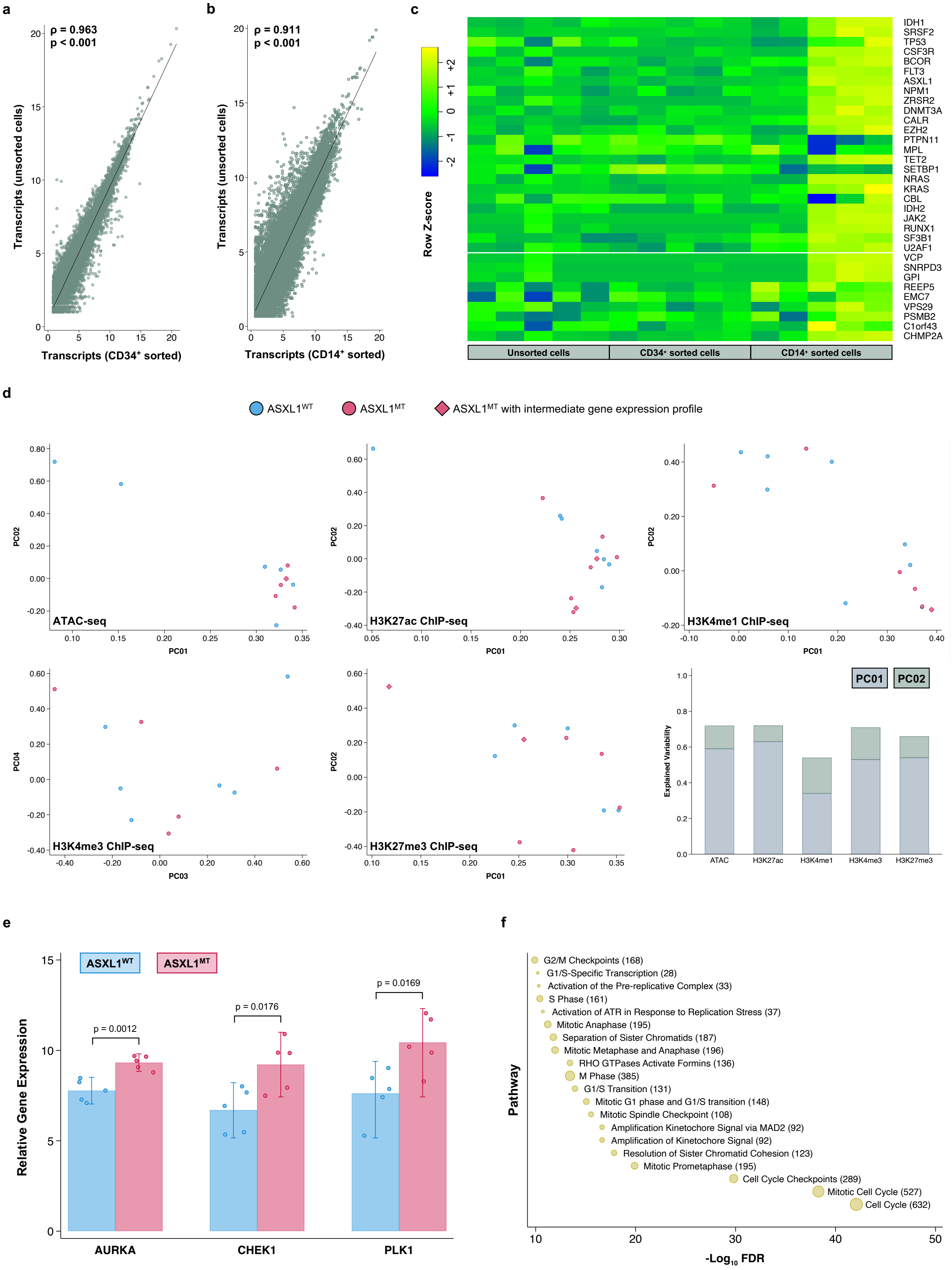

**Supplementary Figure 2** showing the rationale for cell selection and sample classification: RNA-seq of sorted cell populations and principal component analysis of the ChIP-seq and ATAC-seq data. **a**, Scatter plot showing pairwise correlations (Spearman's rank correlation coefficient), demonstrating a strong positive monotonic relationship between the transcript count in CD34-sorted and unsorted cells. **b**, Scatter plot showing pairwise correlations (Spearman's rank correlation coefficient), demonstrating a strong positive monotonic relationship between the transcript count in CD14-sorted and unsorted cells. **c**, Heatmap showing standardized expression levels per gene for select genes of interest. There were no consistent differences between CD34-sorted and unsorted cells for genes relevant to myeloid biology or housekeeping genes. Three of the 5 CD14-sorted patient samples show slightly higher expression values across myeloid and housekeeping genes suggesting sample differences rather than differences inherent to the cell sorting strategy. **d**, Scatter plots showing the first two principal components for each ChIP-seq and ATAC-seq sample included in the chromatin model, stratified by genotype and gene expression profile. The epigenetic profile of the ASXL1$^{MT}$ samples with an intermediate gene expression profile were similar to the remainder of the samples (*a priori* sample classification based on *ASXL1* genotype was maintained for all downstream analyses). **e**, Bar graphs showing the increased expression of the mitotic kinases AURKA, CHEK1, and PLK1 in ASXL1$^{MT}$ CMML by quantitative PCR methodology (targeted validation of key overexpressed transcriptomic targets; n=10 biologically independent samples; unpaired Student's t-test, raw p-values without adjustment for multiple hypothesis testing are shown). Bars represent means, whiskers 95% confidence intervals. **f**, Scatter plot showing the top 20 differentially regulated pathways including key mitotic pathways. The marker size is proportional to the number of genes per cluster (shown in parentheses).

## SUPPLEMENTARY FIGURE 3

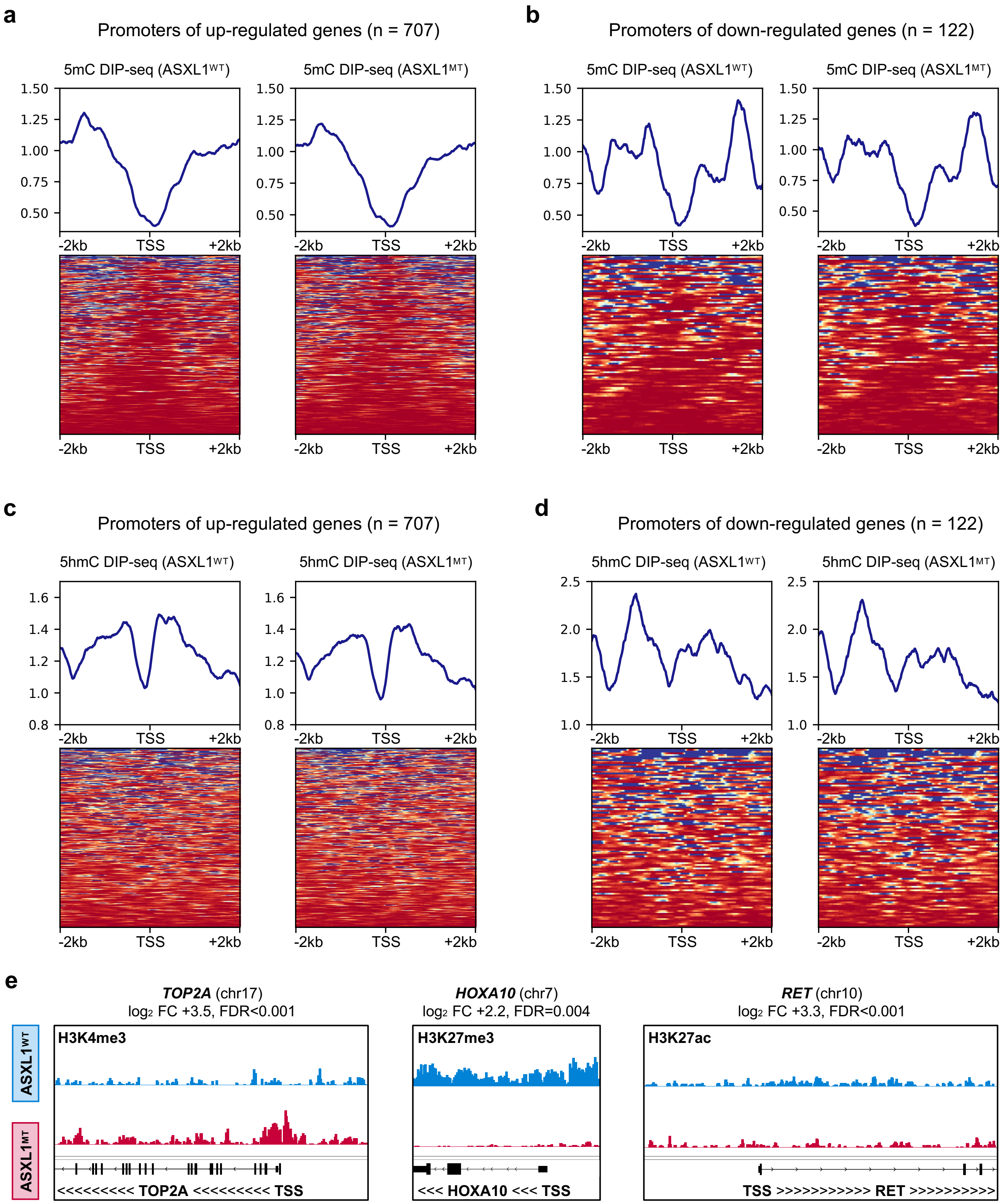

**Supplementary Figure 3** showing the 5mC and 5hmC signals in promoter regions of differentially expressed genes for both *ASXL1* genotypes. **a**, Average signal curves and heatmap representations for 5mC in the promoter regions of the up-regulated genes stratified by *ASXL1* genotype. **b**, Average signal curves and heatmap representations for 5mC in the promoter regions of the down-regulated genes stratified by *ASXL1* genotype. **c**, Average signal curves and heatmap representations for 5hmC in the promoter regions of the up-regulated genes stratified by *ASXL1* genotype. **d**, Average signal curves and heatmap representations for 5hmC in the promoter regions of the down-regulated genes stratified by *ASXL1* genotype. **e**, ChIP-seq signal tracks showing examples of histone modifications in promoter regions of up-regulated genes as referenced in **Figure 3e** (in *ASXL1*-wildtype and -mutant patients).

## SUPPLEMENTARY FIGURE 4

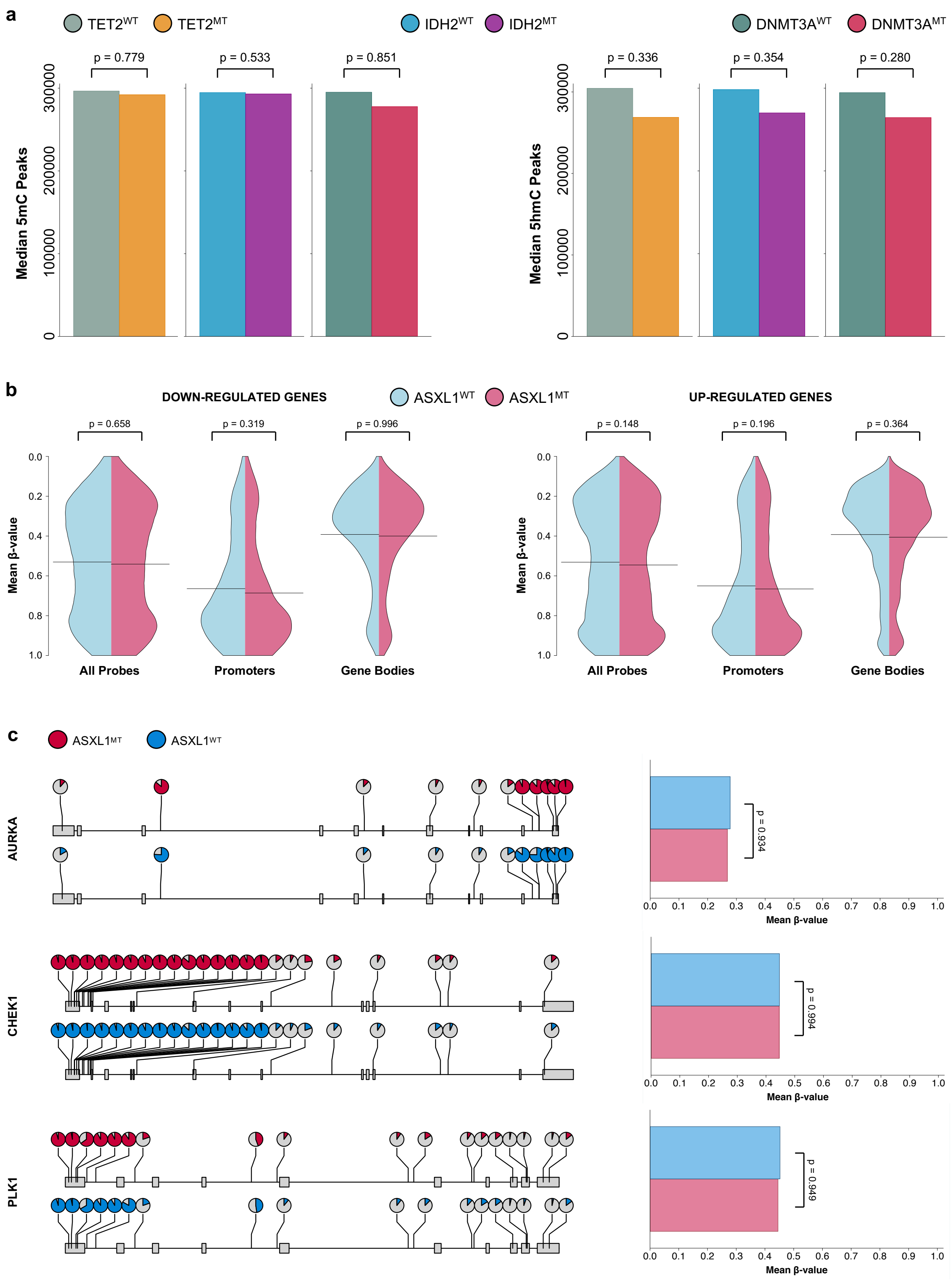

**Supplementary Figure 4** showing the effects of gene body (hydroxy)methylation on gene expression using DIP-seq and microarray studies (validation). **a**, Bar graphs showing the median global number of methylation and hydroxymethylation peaks stratified by potentially confounding co-mutations. There was no evidence of differential methylation or hydroxymethylation when stratifying by the presence of *TET2*, *DNMT3A*, or *IDH2* mutations. **b**, Bean plots showing the mean $\beta$-values for all methylation probes per gene as well as probes in promoters and gene bodies for up- and down-regulated genes separately. **c**, Lollipop plots showing individual probe $\beta$-values for select up-regulated genes of interest for the two genotypes separately. Pie charts represent the degree of methylation ($\beta$-value), bars indicate exons. Bar graphs show the mean $\beta$-value per gene of interest for the two genotypes separately. The two-sided Mann-Whitney U test was used to compared groups in **4a**-**c**, raw p-values without adjustment for multiple hypothesis testing are shown.

## SUPPLEMENTARY FIGURE 5

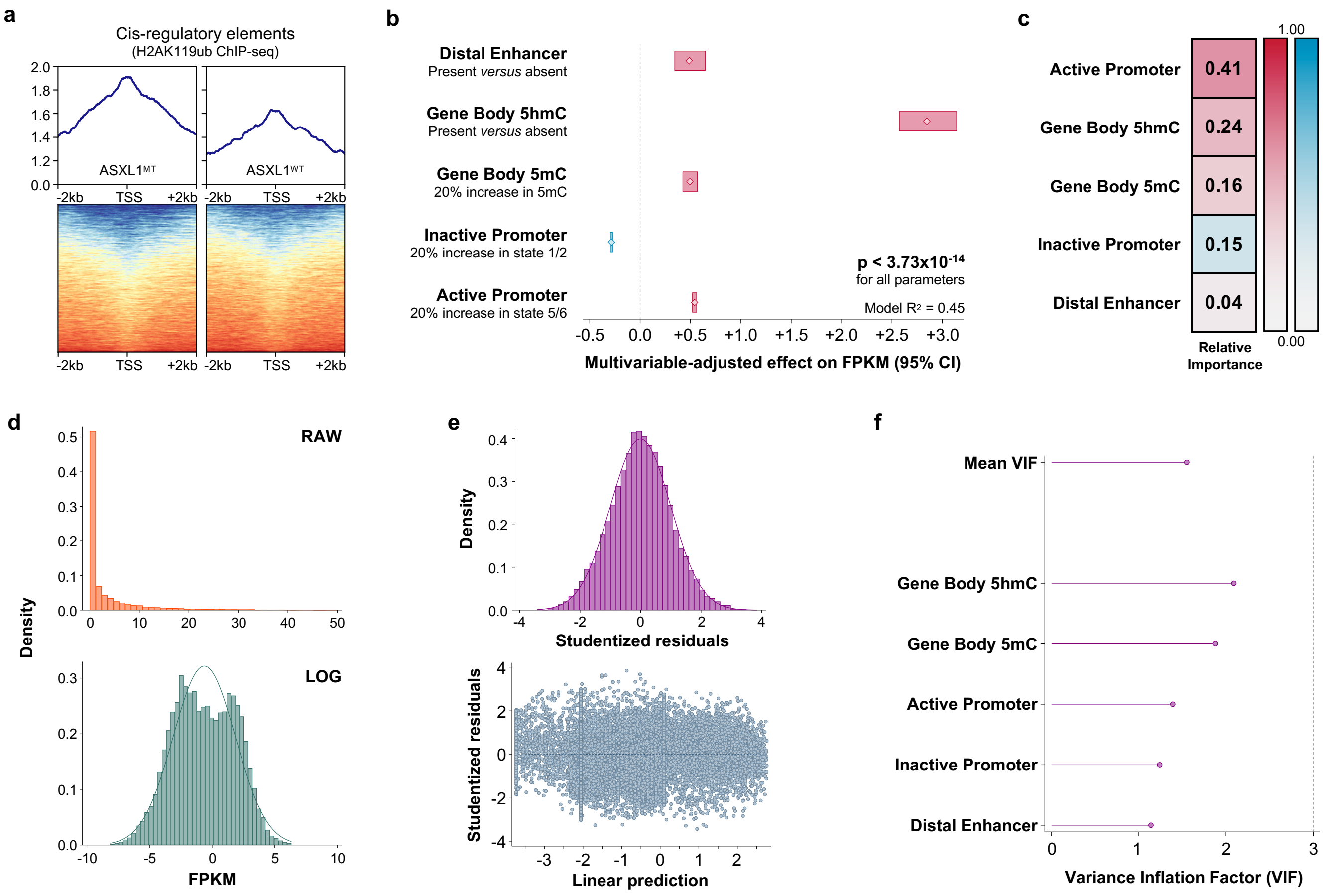

**Supplementary Figure 5** showing the independent associations between promoter chromatin states, distal enhancers, gene body (hydroxy)methylation, and gene expression in ASXL1$^{MT}$ CMML. A measure of the relative importance of these epigenetic regulatory mechanisms for transcriptional activity and relevant regression diagnostics are presented. **a**, Line plots and heatmaps demonstrating the H2AK119ub signal in the identified cis-regulatory regions. **b**, Forest plot showing the multivariable-adjusted (independent) effects of promoter chromatin states, distal enhancers, and gene body (hydroxy)methylation on gene expression in ASXL1$^{MT}$ CMML. The independent effects of the different epigenetic regulatory mechanisms on gene expression (measured by log-transformed FPKM) were estimated using ordinary least squares (OLS) regression. Effect sizes represent relative changes in gene expression (+0.5 corresponds to a 50% increase in gene expression). The model explained 44.9% of the variation in gene expression. **c**, Heatmap showing a measure of the relative importance of each model parameter (hierarchical partitioning of $R^2$). **d**, Histograms and normal kernel density estimates showing effect of log-transformation on the transcript read counts. **e**, Histogram and normal kernel density estimate showing the distribution of the studentized OLS regression residuals. There was no evidence of a gross violation of the normality assumption. Scatter plot showing the studentized residuals against the fitted values. There was no evidence of a gross violation of the homoscedasticity assumption. **f**, Variance inflation factors for the predictors in the OLS regression. There was no evidence of relevant multicollinearity.

# SUPPLEMENTARY FIGURE 6

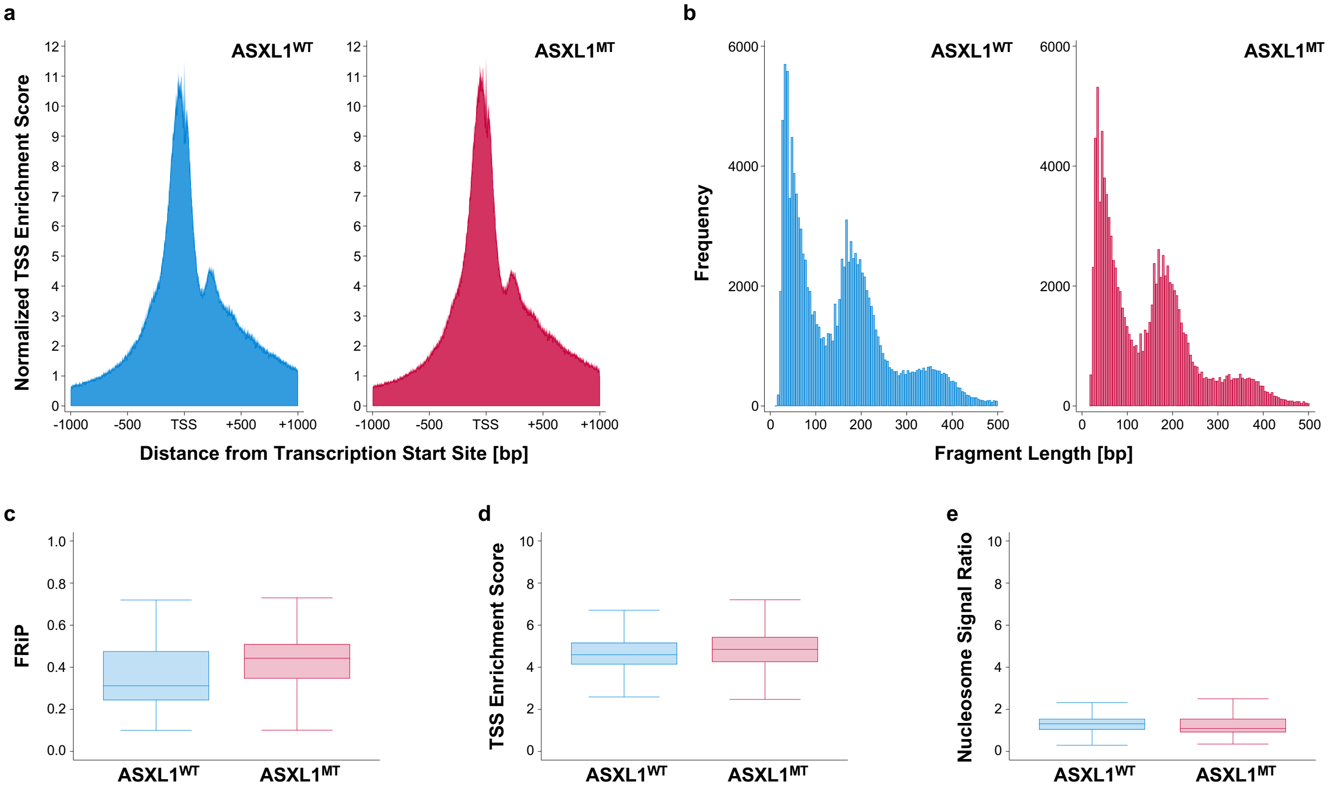

**Supplementary Figure 6** showing single-cell ATAC-seq quality metrics. **a**, Area plots showing the normalized transcription start site (TSS) enrichment scores (ratio of fragments centered at the TSS to fragments in flanking regions) for 12192 single cells from CMML patients (stratified by *ASXL1* genotype). **b**, Histograms showing the fragment length distribution for 12192 single cells from CMML patients (stratified by *ASXL1* genotype). **c**, Box plots showing the fraction of reads in peaks (FRiP) for 12192 single cells from CMML patients (stratified by *ASXL1* genotype). **d**, Box plots showing the TSS enrichment scores for 12192 single cells from CMML patients (stratified by *ASXL1* genotype). **e**, Box plots showing the nucleosome signal ratio (mono-nucleosomal to nucleosome-free fragments) for 12192 single cells from CMML patients (stratified by *ASXL1* genotype). Data are presented as standard Tukey boxplots (with the box encompassing Q1 to Q3, the median denoted as a central horizontal line in the box, and the whiskers covering the data within ±1.5 IQR in **6c**-**e**).
