# Supplementary material for: Oncogenic gene expression and epigenetic remodeling of cis-regulatory elements in ASXL1-mutant chronic myelomonocytic leukemia
Source: Nat Commun. 2022 Mar 17;13:1434. doi: 10.1038/s41467-022-29142-6 (PMC8931048; doi:10.1038/s41467-022-29142-6)
Supplement: Supplementary file 2 — Description of Additional Supplementary Files [file 41467_2022_29142_MOESM2_ESM.pdf]

Description of Additional Supplementary Files

File Name: Supplementary Data 1
Description: Primer sets used for quantitative reverse transcription polymerase chain reaction validation.

File Name: Supplementary Data 2
Description: Characteristics of 16 patients with chronic myelomonocytic leukemia (8 ASXL1-wildtype, 8 ASXL1-mutant).

File Name: Supplementary Data 3
Description: Differential gene expression (ASXL1-mutant versus -wildtype chronic myelomonocytic leukemia).

File Name: Supplementary Data 4
Description: Functional annotation of genes overexpressed in ASXL1-mutant chronic myelomonocytic leukemia (druggable targets).

File Name: Supplementary Data 5
Description: Functional annotation of differentially expressed genes (up- and down-regulated in ASXL1-mutant chronic myelomonocytic leukemia).

File Name: Supplementary Data 6
Description: Pathway analysis of differentially expressed genes between ASXL1-mutant and -wildtype chronic myelomonocytic leukemia.
