# Supplementary material for: Oncogenic gene expression and epigenetic remodeling of cis-regulatory elements in ASXL1-mutant chronic myelomonocytic leukemia
Source: Nat Commun. 2022 Mar 17;13:1434. doi: 10.1038/s41467-022-29142-6 (PMC8931048; doi:10.1038/s41467-022-29142-6)
Supplement: Supplementary file 9 — Reporting Summary [file 41467_2022_29142_MOESM9_ESM.pdf]

nature research

Corresponding author(s): Mrinal M. Patnaik, MD
Alexandre Gaspar Maia, PhD

Last updated by author(s): Feb 23rd, 2022

Reporting Summary

Nature Research wishes to improve the reproducibility of the work that we publish. This form provides structure for consistency and transparency in reporting. For further information on Nature Research policies, see our Editorial Policies and the Editorial Policy Checklist.

Statistics

For all statistical analyses, confirm that the following items are present in the figure legend, table legend, main text, or Methods section.

n/a	Confirmed	
☐	☒	The exact sample size (*n*) for each experimental group/condition, given as a discrete number and unit of measurement
☐	☒	A statement on whether measurements were taken from distinct samples or whether the same sample was measured repeatedly
☐	☒	The statistical test(s) used AND whether they are one- or two-sided<br>*Only common tests should be described solely by name; describe more complex techniques in the Methods section.*
☐	☒	A description of all covariates tested
☐	☒	A description of any assumptions or corrections, such as tests of normality and adjustment for multiple comparisons
☐	☒	A full description of the statistical parameters including central tendency (e.g. means) or other basic estimates (e.g. regression coefficient) AND variation (e.g. standard deviation) or associated estimates of uncertainty (e.g. confidence intervals)
☐	☒	For null hypothesis testing, the test statistic (e.g. *F*, *t*, *r*) with confidence intervals, effect sizes, degrees of freedom and *P* value noted<br>*Give P values as exact values whenever suitable.*
☐	☒	For Bayesian analysis, information on the choice of priors and Markov chain Monte Carlo settings
☒	☐	For hierarchical and complex designs, identification of the appropriate level for tests and full reporting of outcomes
☐	☒	Estimates of effect sizes (e.g. Cohen's *d*, Pearson's *r*), indicating how they were calculated

Our web collection on statistics for biologists contains articles on many of the points above.

Software and code

Policy information about availability of computer code

Data collection	No software was used for data collection.
Data analysis	featureCounts (v2.0.0), DESeq2 (v1.30.1), heatmap.2 (v3.1.1), EnhancedVolcano (v1.8.0), gProfiler (v2021-05-01), Reactome Pathway Knowledgebase (v2021-5-7), DGIdb (v3.0), bowtie2 (v2.3.3.1), Minfi (v1.36.0), bumphunter (v1.32.0), Cell Ranger ATAC software (v2.0), samtools (v1.9), MACS2 (v3.0.0a6), MSPC (v5.4.0), deepTools (v3.5.0), wiggletools (v1.2), ChromHMM (v1.22), GREAT (v4.0.4), HOMER (v3.0), Seurat (v4.0.5), Harmony (v0.1.0), BioQC (v1.18.0), R (v4.0.5), Stata (v16.1). References for all software packages and key parameters are listed in the Methods section.

For manuscripts utilizing custom algorithms or software that are central to the research but not yet described in published literature, software must be made available to editors and reviewers. We strongly encourage code deposition in a community repository (e.g. GitHub). See the Nature Research guidelines for submitting code & software for further information.

Data

Policy information about availability of data

All manuscripts must include a data availability statement. This statement should provide the following information, where applicable:

- Accession codes, unique identifiers, or web links for publicly available datasets
- A list of figures that have associated raw data
- A description of any restrictions on data availability

The raw RNA sequencing data generated in this study have been deposited in the Gene Expression Omnibus (GEO) database under accession code GSE159543 [https://www.ncbi.nlm.nih.gov/geo/query/acc.cgi?acc=GSE159543]. The raw ChIP, DIP, and ATAC sequencing data generated in this study have been deposited in the GEO database under accession code GSE159886 [https://www.ncbi.nlm.nih.gov/geo/query/acc.cgi?acc=GSE159886]. The remaining data are available within the Article, Supplementary Information, or Source Data files. Source data are provided with this paper for Figure 1d and Figure 6b-d. The publicly available

transcription factor ChIP sequencing data used in this study are available in the ReMap 2020 database [https://remap2020.univ-amu.fr]. The publicly available data on candidate cis-regulatory elements used in this study are available through the ENCODE database [https://www.encodeproject.org], the GeneHancer tracks in the UCSC Genome Browser [https://genome.ucsc.edu], and the Hacer database [http://bioinfo.vanderbilt.edu/AE/HACER/].

Field-specific reporting

Please select the one below that is the best fit for your research. If you are not sure, read the appropriate sections before making your selection.

☒ Life sciences ☐ Behavioural & social sciences ☐ Ecological, evolutionary & environmental sciences

For a reference copy of the document with all sections, see nature.com/documents/nr-reporting-summary-flat.pdf

Life sciences study design

All studies must disclose on these points even when the disclosure is negative.

Sample size

The sample size of 8 per group was chosen based on the RNA-seq power calculation (at least 80% power to detect a 2-fold or greater change in gene expression with 7 samples per group). The factors determining the required sample size (n) for this experimental design are the desired power (set as ≥ 80.0%, considered acceptable by the investigator), the α-level (set as 0.05, considered acceptable by the investigator), the effect size (ES, set as ≥ 2-fold change in gene expression for a given gene, considered biologically significant by the investigator), the average sequencing coverage (CO, set as ≥ 20, empirically derived from previous results using the same sequencing facility and platform), and the coefficient of variation (CV, set as 0.4, empirically derived from a large number of human RNA-seq experiments, please see Hart et al. J Comput Biol 2013;20(12):970-978).

Data exclusions

Pre-defined quality control thresholds were used for data collection and experiments. Twenty-two of 160 samples (13.8%) were excluded based on quality control measures (sample preparation, contamination, inadequate sequencing depth, or limited ChIP enrichment). An explanation of these exclusions can be found in Supplementary Figure 1c.

Replication

After applying the quality control measures mentioned above at least 5 biological replicates per group remained for all omics layers and comparisons. Key findings from RNA-seq were confirmed by RT-qPCR (n = 10, performed in triplicate). Successful experiments for biological replicates included: DNA (n=16/16, 100% successful), RNA (n=14/16, 88% successful), ChIP-seq (n=67/80, 84% successful), DIP-seq (n=30/32, 84% successful), and ATAC-seq (n=11/16, 69% successful). Key findings from bulk ATAC-seq were confirmed by single-cell ATAC-seq (n=12192 single cells).

Randomization

This was not a randomized experiment. Experimental groups were defined by genotype a priori for all analyses.

Blinding

N/A. The scientists performing the experiments were unaware of the genotype of the samples at the time of data generation. The scientists performing data analysis were aware of the genotype of the samples as required to carry out the group comparisons of interest.

Reporting for specific materials, systems and methods

We require information from authors about some types of materials, experimental systems and methods used in many studies. Here, indicate whether each material, system or method listed is relevant to your study. If you are not sure if a list item applies to your research, read the appropriate section before selecting a response.

Materials & experimental systems

n/a	Involved in the study
☐	☒ Antibodies
☒	☐ Eukaryotic cell lines
☒	☐ Palaeontology and archaeology
☒	☐ Animals and other organisms
☐	☒ Human research participants
☒	☐ Clinical data
☒	☐ Dual use research of concern

Methods

n/a	Involved in the study
☐	☒ ChIP-seq
☒	☐ Flow cytometry
☒	☐ MRI-based neuroimaging

Antibodies

Antibodies used

H3K4me1-C15410037 | Catalogue Number C15410037-50 (Diagenode, Denville, United States)
H3K4me3-9751S | Catalogue Number C15410003-50 (Cell Signaling Technology, Danvers, United States)
H3K27me3-pAb-069-050 | Catalogue Number C15410069 (Diagenode, Denville, United States)
5mC-33D3 | Catalogue Number C15200006-100 (Diagenode, Denville, United States)
5hmC | In-house developed (Mayo Clinic Epigenomics Development Laboratory, Rochester, United States)
IgG Bridging Antibody | Catalogue Number 53017 (Active Motif, Carlsbad, United States)
H2AK119ub-D27C4 | Catalogue Number 8240T (Cell Signaling Technology, Danvers, United States)
H3K27ac-D5E4 | Catalogue Number 8173T (Cell Signaling Technology, Danvers, United States)

	The antibodies for ChIP-seq were used at a dilution of 1:250 (H3K27me3), 1:500 (H3K4me1 and H3K4me3), and 1:1000 (H2AK119ub and H3K27ac). The antibodies for DIP-seq were used at a dilution of 1:110 (5hmC) and 1:340 (5mC).
Validation	Validation of all antibodies was performed by the respective manufacturers they were purchased from.<br><br>For the Diagenode, Active Motif, and Cell Signaling antibodies, detailed validation data can be found under the respective catalogue numbers on the manufacturers' websites (https://www.diagenode.com/en/p/h3k4me1-polyclonal-antibody-classic-50-ug-18-ul, https://www.diagenode.com/en/p/h3k4me3-polyclonal-antibody-premium-50-ug-50-ul, https://www.diagenode.com/en/p/h3k27me3-polyclonal-antibody-classic-50-mg-34-ml, https://www.diagenode.com/en/p/5-mc-monoclonal-antibody-cl-b-classic-100-ug-50-ul, https://www.activemotif.com/catalog/494/bridging-antibody-for-mouse-igg, https://www.cellsignal.com/products/primary-antibodies/ubiquityl-histone-h2a-lys119-d27c4-xp-rabbit-mab/8240, https://www.cellsignal.com/products/primary-antibodies/acetyl-histone-h3-lys27-d5e4-xp-rabbit-mab/8173). For the in-house developed 5hmC antibody, detailed validation data can be found in Tiedemann et al. Cell Rep 2014;9(4):1554-1566.

Human research participants

Policy information about studies involving human research participants

Population characteristics	The population characteristics as well as selection criteria are described in detail in the manuscript (Table 1).
Recruitment	The patients were recruited from the Mayo Clinic CMML Biorepository (IRB-15-003786). Informed consent was obtained from all patients in accordance with the Declaration of Helsinki. Since Chronic Myelomonocytic Leukemia is a rare disease and Mayo Clinic a tertiary referral center, the presence of a referral bias cannot be excluded (patient characteristics such as performance status and socioeconomic factors may influence a patient's ability to travel to the referral center to be evaluated). The results of this study may therefore reflect the biology of patients willing and able to be evaluated at a tertiary referral center.
Ethics oversight	Mayo Clinic Institutional Review Board (IRB-15-003786).

Note that full information on the approval of the study protocol must also be provided in the manuscript.

ChIP-seq

Data deposition

☒ Confirm that both raw and final processed data have been deposited in a public database such as GEO.

☒ Confirm that you have deposited or provided access to graph files (e.g. BED files) for the called peaks.

Data access links<br>*May remain private before publication.*	GSE159543 (https://www.ncbi.nlm.nih.gov/geo/query/acc.cgi?acc=GSE159543)<br>GSE159886 (https://www.ncbi.nlm.nih.gov/geo/query/acc.cgi?acc=GSE159886)
Files in database submission	GSM4832243 RNA-seq Sample 01<br>GSM4832244 RNA-seq Sample 02<br>GSM4832245 RNA-seq Sample 03<br>GSM4832246 RNA-seq Sample 04<br>GSM4832247 RNA-seq Sample 05<br>GSM4832248 RNA-seq Sample 06<br>GSM4832249 RNA-seq Sample 07<br>GSM4832250 RNA-seq Sample 08<br>GSM4832251 RNA-seq Sample 09<br>GSM4832252 RNA-seq Sample 10<br>GSM4832253 RNA-seq Sample 11<br>GSM4832254 RNA-seq Sample 12<br>GSM4832255 RNA-seq Sample 13<br>GSM4832256 RNA-seq Sample 14<br>GSE159543 Raw Feature Counts<br>GSM4849396 5hmC Sample 01<br>GSM4849396 5hmC Sample 02<br>GSM4849396 5hmC Sample 03<br>GSM4849396 5hmC Sample 04<br>GSM4849396 5hmC Sample 05<br>GSM4849396 5hmC Sample 06<br>GSM4849396 5hmC Sample 07<br>GSM4849396 5hmC Sample 08<br>GSM4849396 5hmC Sample 09<br>GSM4849396 5hmC Sample 10<br>GSM4849396 5hmC Sample 11<br>GSM4849396 5hmC Sample 12<br>GSM4849396 5hmC Sample 13

GSM4849396 5hmC Sample 14
GSM4849396 5hmC Sample 16
GSM4849396 5mC Sample 01
GSM4849396 5mC Sample 02
GSM4849396 5mC Sample 03
GSM4849396 5mC Sample 04
GSM4849396 5mC Sample 05
GSM4849396 5mC Sample 06
GSM4849396 5mC Sample 07
GSM4849396 5mC Sample 08
GSM4849396 5mC Sample 09
GSM4849396 5mC Sample 10
GSM4849396 5mC Sample 11
GSM4849396 5mC Sample 12
GSM4849396 5mC Sample 13
GSM4849396 5mC Sample 14
GSM4849396 5mC Sample 16
GSM4849396 ATAC Sample 03
GSM4849396 ATAC Sample 04
GSM4849396 ATAC Sample 05
GSM4849396 ATAC Sample 06
GSM4849396 ATAC Sample 07
GSM4849396 ATAC Sample 08
GSM4849396 ATAC Sample 09
GSM4849396 ATAC Sample 12
GSM4849396 ATAC Sample 13
GSM4849396 ATAC Sample 15
GSM4849396 ATAC Sample 16
GSM4849396 H2AK119ub Sample 01
GSM4849396 H2AK119ub Sample 02
GSM4849396 H2AK119ub Sample 03
GSM4849396 H2AK119ub Sample 04
GSM4849396 H2AK119ub Sample 06
GSM4849396 H2AK119ub Sample 07
GSM4849396 H2AK119ub Sample 08
GSM4849396 H2AK119ub Sample 09
GSM4849396 H2AK119ub Sample 10
GSM4849396 H2AK119ub Sample 11
GSM4849396 H2AK119ub Sample 12
GSM4849396 H2AK119ub Sample 13
GSM4849396 H2AK119ub Sample 14
GSM4849396 H2AK119ub Sample 15
GSM4849396 H2AK119ub Sample 16
GSM4849396 H3K4me1 Sample 01
GSM4849396 H3K4me1 Sample 02
GSM4849396 H3K4me1 Sample 03
GSM4849396 H3K4me1 Sample 04
GSM4849396 H3K4me1 Sample 05
GSM4849396 H3K4me1 Sample 06
GSM4849396 H3K4me1 Sample 07
GSM4849396 H3K4me1 Sample 08
GSM4849396 H3K4me1 Sample 09
GSM4849396 H3K4me1 Sample 11
GSM4849396 H3K4me1 Sample 12
GSM4849396 H3K4me1 Sample 13
GSM4849396 H3K4me1 Sample 15
GSM4849396 H3K4me1 Sample 16
GSM4849396 H3K4me3 Sample 01
GSM4849396 H3K4me3 Sample 02
GSM4849396 H3K4me3 Sample 03
GSM4849396 H3K4me3 Sample 04
GSM4849396 H3K4me3 Sample 05
GSM4849396 H3K4me3 Sample 06
GSM4849396 H3K4me3 Sample 10
GSM4849396 H3K4me3 Sample 12
GSM4849396 H3K4me3 Sample 14
GSM4849396 H3K4me3 Sample 15
GSM4849396 H3K4me3 Sample 16

GSM4849396 H3K27ac Sample 01
GSM4849396 H3K27ac Sample 02
GSM4849396 H3K27ac Sample 03
GSM4849396 H3K27ac Sample 04
GSM4849396 H3K27ac Sample 05
GSM4849396 H3K27ac Sample 06
GSM4849396 H3K27ac Sample 07
GSM4849396 H3K27ac Sample 08
GSM4849396 H3K27ac Sample 09
GSM4849396 H3K27ac Sample 10
GSM4849396 H3K27ac Sample 11
GSM4849396 H3K27ac Sample 12
GSM4849396 H3K27ac Sample 13
GSM4849396 H3K27ac Sample 14
GSM4849396 H3K27ac Sample 15
GSM4849396 H3K27me3 Sample 02
GSM4849396 H3K27me3 Sample 03
GSM4849396 H3K27me3 Sample 04
GSM4849396 H3K27me3 Sample 06
GSM4849396 H3K27me3 Sample 07
GSM4849396 H3K27me3 Sample 11
GSM4849396 H3K27me3 Sample 12
GSM4849396 H3K27me3 Sample 13
GSM4849396 H3K27me3 Sample 14
GSM4849396 H3K27me3 Sample 15
GSM4849396 H3K27me3 Sample 16
GSM4849396 5mC 5hmC Input Sample 01
GSM4849396 H3K27ac H2AK119ub Input Sample 01
GSM4849396 H3K4me1 H3K4me3 H3K27me3 Input Sample 01
GSM4849396 5mC 5hmC Input Sample 02
GSM4849396 H3K27ac H2AK119ub Input Sample 02
GSM4849396 H3K4me1 H3K4me3 H3K27me3 Input Sample 02
GSM4849396 5mC 5hmC Input Sample 03
GSM4849396 H3K27ac H2AK119ub Input Sample 03
GSM4849396 H3K4me1 H3K4me3 H3K27me3 Input Sample 03
GSM4849396 5mC 5hmC Input Sample 04
GSM4849396 H3K27ac H2AK119ub Input Sample 04
GSM4849396 H3K4me1 H3K4me3 H3K27me3 Input Sample 04
GSM4849396 5mC 5hmC Input Sample 05
GSM4849396 H3K4me1 H3K4me3 H3K27me3 Input Sample 05
GSM4849396 5mC 5hmC Input Sample 06
GSM4849396 H3K27ac H2AK119ub Input Sample 06
GSM4849396 H3K4me1 H3K4me3 H3K27me3 Input Sample 06
GSM4849396 5mC 5hmC Input Sample 07
GSM4849396 H3K27ac H2AK119ub Input Sample 07
GSM4849396 H3K4me1 H3K4me3 H3K27me3 Input Sample 07
GSM4849396 5mC 5hmC Input Sample 08
GSM4849396 H3K27ac H2AK119ub Input Sample 08
GSM4849396 H3K4me1 H3K4me3 H3K27me3 Input Sample 08
GSM4849396 5mC 5hmC Input Sample 09
GSM4849396 H3K27ac H2AK119ub Input Sample 09
GSM4849396 H3K4me1 H3K4me3 H3K27me3 Input Sample 09
GSM4849396 5mC 5hmC Input Sample 10
GSM4849396 H3K27ac H2AK119ub Input Sample 10
GSM4849396 H3K4me1 H3K4me3 H3K27me3 Input Sample 10
GSM4849396 5mC 5hmC Input Sample 11
GSM4849396 H3K27ac H2AK119ub Input Sample 11
GSM4849396 H3K4me1 H3K4me3 H3K27me3 Input Sample 11
GSM4849396 5mC 5hmC Input Sample 12
GSM4849396 H3K27ac H2AK119ub Input Sample 12
GSM4849396 H3K4me1 H3K4me3 H3K27me3 Input Sample 12
GSM4849396 5mC 5hmC Input Sample 13
GSM4849396 H3K27ac H2AK119ub Input Sample 13
GSM4849396 H3K4me1 H3K4me3 H3K27me3 Input Sample 13
GSM4849396 5mC 5hmC Input Sample 14
GSM4849396 H3K27ac H2AK119ub Input Sample 14
GSM4849396 H3K4me1 H3K4me3 H3K27me3 Input Sample 14
GSM4849396 H3K27ac H2AK119ub Input Sample 14

GSM4849396 H3K4me1 H3K4me3 H3K27me3 Input Sample 15
GSM4849396 5mC 5hmC Input Sample 16
GSM4849396 H3K27ac H2AK119ub Input Sample 16
GSM4849396 H3K4me1 H3K4me3 H3K27me3 Input Sample 16
GSM4849396 ASXL1 MT 5hmC Consensus Peaks
GSM4849396 ASXL1 MT 5mC Consensus Peaks
GSM4849396 ASXL1 MT ATAC Consensus Peaks
GSM4849396 ASXL1 MT ChromHMM States
GSM4849396 ASXL1 MT H3K27ac Consensus Peaks
GSM4849396 ASXL1 MT H3K27me3 Consensus Peaks
GSM4849396 ASXL1 MT H3K4me1 Consensus Peaks
GSM4849396 ASXL1 MT H3K4me3 Consensus Peaks
GSM4849396 ASXL1 MT H2AK119ub Consensus Peaks
GSM4849396 ASXL1 WT 5hmC Consensus Peaks
GSM4849396 ASXL1 WT 5mC Consensus Peaks
GSM4849396 ASXL1 WT ATAC Consensus Peaks
GSM4849396 ASXL1 WT ChromHMM States
GSM4849396 ASXL1 WT H3K27ac Consensus Peaks
GSM4849396 ASXL1 WT H3K27me3 Consensus Peaks
GSM4849396 ASXL1 WT H3K4me1 Consensus Peaks
GSM4849396 ASXL1 WT H3K4me3 Consensus Peaks
GSM4849396 ASXL1 WT H2AK119ub Consensus Peaks

Genome browser session
(e.g. UCSC)

N/A

Methodology

Replicates

At least 5 biological replicates per group (please see Supplementary Figure 1c).

Sequencing depth

Sequencing was performed on Illumina HiSeq 2500/4000 instruments (Mayo Clinic Sequencing Core standards / quality thresholds).

Antibodies

H3K4me1-C15410037 | Catalogue Number C15410037-50 (Diagenode, Denville, United States)
H3K4me3-9751S | Catalogue Number C15410003-50 (Cell Signaling Technology, Danvers, United States)
H3K27me3-pAb-069-050 | Catalogue Number C15410069 (Diagenode, Denville, United States)
5mC-33D3 | Catalogue Number C15200006-100 (Diagenode, Denville, United States)
5hmC | In-house developed (Mayo Clinic Epigenomics Development Laboratory, Rochester, United States)
IgG Bridging Antibody | Catalogue Number 53017 (Active Motif, Carlsbad, United States)
H2AK119ub-D27C4 | Catalogue Number 8240T (Cell Signaling Technology, Danvers, United States)
H3K27ac-D5E4 | Catalogue Number 8173T (Cell Signaling Technology, Danvers, United States)

The antibodies for ChIP-seq were used at a dilution of 1:250 (H3K27me3), 1:500 (H3K4me1 and H3K4me3), and 1:1000 (H2AK119ub and H3K27ac). The antibodies for DIP-seq were used at a dilution of 1:110 (5hmC) and 1:340 (5mC).

Peak calling parameters

Peaks were called using MACS2 with a standard q-value cutoff of 0.05 in the first step. We then employed a stringent second consensus peak calling step with MSPC (weak threshold p < 1.00x10^-4, stringency threshold p < 1.00x10^-8).

Data quality

Data quality pre- and post-alignment was evaluated with FastQC. Duplicate reads were removed prior to alignment. Truncated, unpaired, and chimeric reads were discarded before proceeding with downstream analyses.

Software

samtools (v1.9), bowtie2 (v2.3.3.1), MACS2 (v3.0.0a6), MSPC (v5.4.0), deepTools (v3.5.0), wiggletools (v1.2), ChromHMM (v1.22), R (v4.0.5). References for all software packages and key parameters are listed in the Methods section.
